# Fabrication of CNT-N@Manganese Oxide Hybrid Nanomaterials through a Versatile One-Pot Eco-Friendly Route toward Engineered Textile Supercapacitors

**DOI:** 10.1021/acsaenm.4c00164

**Published:** 2024-03-29

**Authors:** Joana S. Teixeira, Rui S. Costa, Alexandra Guedes, André M. Pereira, Clara R. Pereira

**Affiliations:** † REQUIMTE/LAQV, Departamento de Química e Bioquímica, Faculdade de Ciências, Universidade do Porto, Rua do Campo Alegre s/n, 4169-007 Porto, Portugal; ‡ IFIMUP, Instituto de Física de Materiais Avançados, Nanotecnologia e Fotónica, Departamento de Física e Astronomia, Faculdade de Ciências, Universidade do Porto, Rua do Campo Alegre s/n, 4169-007 Porto, Portugal; § Instituto de Ciências da Terra − Pólo Porto, Departamento de Geociências, Ambiente e Ordenamento do Território, Faculdade de Ciências, Universidade do Porto, Rua do Campo Alegre s/n, 4169-007 Porto, Portugal

**Keywords:** N-doped carbon nanotubes, manganese oxide, aqueous precipitation process, hybrid textile supercapacitor, energy storage

## Abstract

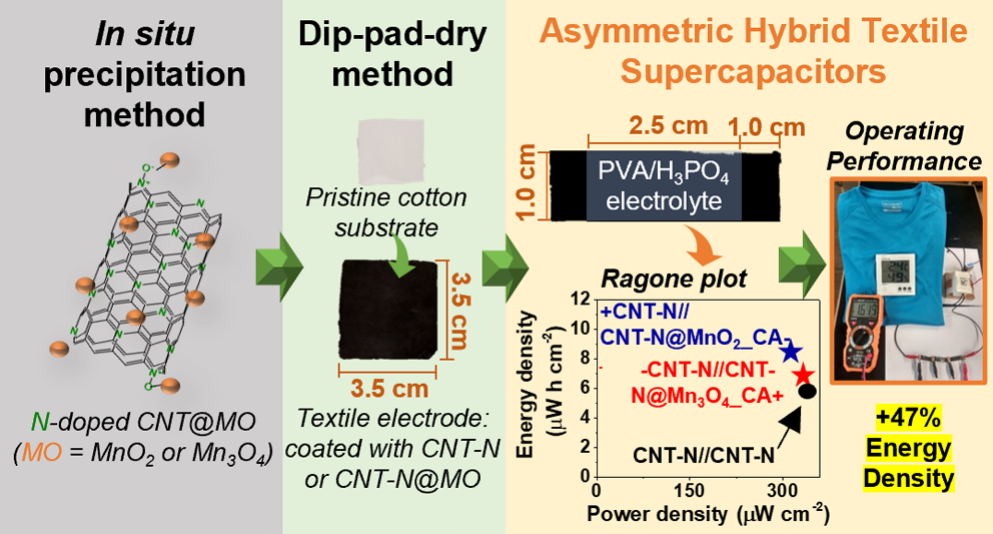

The expansion of the Internet of Things market and the proliferation of wearable technologies have generated a significant demand for textile-based energy storage systems. This work reports the engineered design of hybrid electrode nanomaterials of N-doped carbon nanotubes (CNT-N) functionalized with two types of manganese oxides (MOs)—birnessite (MnO_2_) and hausmannite (Mn_3_O_4_)—and their application in solid-state textile-based hybrid supercapacitors (SCs). A versatile citric acid-mediated eco-friendly one-pot aqueous precipitation process is proposed for the fabrication of the hybrids. Remarkably, different types of MOs were obtained by simply changing the reaction temperature from room temperature to 100 °C, without any *post*-thermal treatment. Asymmetric textile SCs were developed using cotton fabrics coated with CNT-N and the hybrids as textile electrodes, and poly(vinyl) alcohol/orthophosphoric acid as the solid-gel electrolyte. The asymmetric devices presented enhanced energy storage performance relative to the symmetric device based on CNT-N and excellent cycling stability (>96%) after 8000 charge/discharge cycles owing to synergistic effects between CNT-N and the MOs, which endowed nonfaradaic and pseudocapacitive features to the SCs. The asymmetric SC based on CNT-N@MnO_2_ featured 47% higher energy density and comparable power density to the symmetric CNT-N-based device (8.70 W h cm^–2^ at 309.01 μW cm^–2^ vs. 5.93 W h cm^–2^ at 346.58 μW cm^–2^). The engineered hybrid CNT-N@MO nanomaterials and the eco-friendly citric acid-assisted one-pot precipitation route open promising prospects not only for energy storage, but also for (photo)(electro)catalysis, wastewater treatment, and (bio)sensing.

## 1 Introduction

The fast technological evolution has sparked a revolution in electronic components toward more flexible, compact, and lighter smart devices.^1^ In the era of the Internet of Things, smart electronic textiles (e-textiles) became the focus of wide interest for diversified applications, including healthcare, sportswear, fashion, defense, environmental monitoring, among other.^1,2^

The emergence of wearable e-textiles triggered the development of innovative energy storage solutions able to power sensors and flexible displays integrated on clothing.^1,2^ Within energy storage technologies, lithium-ion batteries are the most popular option available in the market to power electronic devices due to their high energy density. However, they present limited cycle life and moderate power density, being also mechanically rigid, restricting their applicability for wearable electronics.^3^ Moreover, the use of lithium continues to be a major concern to comply with safety and environmental requirements.^3^ Supercapacitors (SCs) have been receiving widespread attention as conventional battery substitutes due to their faster charging, higher power density, longer cycle life, and outstanding cyclic stability.^4^ Furthermore, they can be developed on flexible substrates (*e.g*., flexible plastics, elastomeric and textile substrates), being highly attractive for emerging thin and lightweight electronic technologies.^1,4^ Nevertheless, SCs present limited energy density when compared to lithium-ion batteries.^1,4^

The performance of conventional SCs can be enhanced through the engineered design of the active electrode material, in order to conjugate high specific surface area, electrical conductivity, and redox properties.^4^ Carbon-based materials (*e.g*., activated carbon, graphene, carbon quantum dots, carbon nanotubes, carbon fibers/cloth, carbon foams, carbon aerogels, carbon nitrides) and, more recently, conductive metal–organic frameworks (MOFs) and MXenes have been applied as electrode materials in SCs due to their fascinating textural and electrochemical properties.^2,4−8^ In particular, carbon-based materials have been widely used due to their high chemical and mechanical robustness, low known toxicity, tunable porosity, high availability, and versatile modification through oxidation, doping, or functionalization processes, playing a key role in unlocking the potential of SCs for sustainable energy storage applications.^9^ Within this class of nanomaterials, multiwalled carbon nanotubes (MWCNTs) are highlighted, due to their unique tubular structure, large specific surface area, high electrical conductivity (1000–2000 S cm^–1^), and excellent thermal and chemical stabilities.^10^ Despite these advantages, SCs based on carbon nanotubes (CNTs) typically present limited energy density as the electrode material only endows a nonfaradaic energy storage mechanism to the device (electric double-layer capacitors, EDLCs).^5^ The heteroatom doping of CNTs, specifically with nitrogen, which is a strong electron donor, has been pursued, in order to improve their electrical properties and hydrophilicity, also creating new electroactive functional groups.^10^

The hybridization of electrically conductive carbon-based materials with redox-active transition metal oxides is a promising strategy to improve the energy storage performance of SCs, since the resulting hybrid electrode materials can lead to the occurrence of both nonfaradaic and faradaic energy storage mechanisms within the device.^11^ Manganese oxides have been in the spotlight for such hybridization as they are among the most promising surface-redox pseudocapacitive electrode materials for supercapacitors, when considering nontoxicity, electrochemical attributes, and cost.^12^ In particular, birnessite (MnO_2_) and hausmannite (Mn_3_O_4_) electrode materials are highlighted due to their eco-friendliness, diversity of oxidation states of manganese cations (+2, +3 and/or +4), and possible crystallization in different types of morphologies.^12^ The intrinsic supercapacitive performance of MnO_2_ as electrode material has been studied in aqueous neutral and alkaline electrolytes (*e.g*., KOH, Na_2_SO_4_).^13^ Moreover, its importance in the fabrication of SC devices has been reported using electrically conductive substrates (*e.g*., carbon cloth, graphene fibers, Ni foam) and solid-gel electrolytes, including poly(vinyl alcohol) (PVA) combined with KOH, LiClO_4_, H_2_SO_4_, and Na_2_SO_4_.^13^ Similarly, the supercapacitive properties of Mn_3_O_4_ as the electrode material have been discussed in the literature, albeit to a lesser extent, being mainly reported when supported on conductive substrates (*e.g*., stainless steel, carbon fiber, graphene sheet, or Ni foam), in a three-electrode setup; Na_2_SO_4_ aqueous electrolyte has been commonly employed for electrochemical evaluation purposes.^14^ Despite the abovementioned advantages, many challenges are encountered for the practical application of manganese oxides in SCs, which include their low electronic conductivity (10^–5^–10^–6^ S cm^–1^) and chemical robustness.^12^

Thus, the hybridization of CNTs with manganese oxides allows overcoming the intrinsic limitations of both classes of materials, leading to synergetic energy storage properties resulting from the combination of both components.^15^ In the literature, most works in this field of flexible/wearable energy storage devices used commercial or oxidized CNTs in the development of the hybrids and required electrically conductive flexible substrates, such as CNT yarns, carbon fibers, carbon cloth, graphite, and nickel foils.^16−18^ The use of nonconductive daily textiles, such as cotton fabrics, as substrates for the development of flexible/wearable SCs based on carbon@manganese oxide hybrid electrode nanomaterials is limited, despite their widespread availability and accessibility, cost-effectiveness, and easy surface modification for industrial applications. To the best of our knowledge, only Yun et al. reported the development of a symmetric textile-based SC using MnO_2_ nanoparticles (NPs) electrochemically deposited on cotton textiles previously coated with single-walled CNTs (SWCNTs) as electrodes, separated by an insulating polymer textile layer and using Na_2_SO_4_ aqueous solution (1 M) as the electrolyte.^19^ The resulting device presented 2.3× higher energy density when compared with a similar device based on SWCNTs, albeit a 1.3× decrease in the power density. Nevertheless, for wearable applications, the use of liquid electrolytes is a limitation. To date, to the best of our knowledge, there is no work reported in the literature on the design of all-textile-based SCs using nonconductive fabrics as substrates and MWCNTs hybridized with manganese oxides as active electrode materials. In particular, the use of natural cotton fabrics as substrates for SCs offers unique advantages in the context of wearable electronics and smart textiles. Their flexibility, lightness, and widespread availability allow their use in innovative energy storage solutions that can be seamlessly integrated into our daily routine.

This work spotlights the engineered design of novel hybrid electrode nanomaterials composed of N-doped MWCNTs functionalized with manganese oxide (MO) NPs with tailored phase and morphology through a versatile and eco-friendly precipitation route mediated by a citric acid chelating agent. Moreover, the application of the resulting electrode nanomaterials in the development of solid-state textile SCs is spotlighted using a natural textile fabric as the substrate. Prior to the *in situ* immobilization of MO nanomaterials, the MWCNTs were doped with nitrogen-based groups through a green solid-state mechanochemical route (ball milling) in order to tune their electrical properties and create new active sites for the immobilization of MOs. Remarkably, the type of grafted MO phase could be changed from MnO_2_ to Mn_3_O_4_ by simply adjusting the reaction temperature from room temperature to 100 °C during the *in situ* precipitation process without requiring any *post*-thermal treatment step. This feature contrasts to conventional synthesis methods reported in the literature that typically require higher reaction temperatures or thermal *post*-treatments.

The hybrid materials were incorporated onto cotton textile fabrics through the dip-pad-dry process and used as electrodes in sandwich-type asymmetric textile SCs. PVA/H_3_PO_4_ was used as the solid-gel electrolyte, overcoming the limitations of the use of liquid electrolytes (leakage and integrity problems).^20^ A complete electrochemical study of the solid-state hybrid textile SCs was performed in order to unveil the most efficient energy storage system. The influence of the type of Mn_*x*_O_*y*_ material (birnessite vs. hausmannite) grafted to the N-doped MWCNTs on the energy storage outputs of the resulting textile SCs was assessed.

## 2 Experimental Section

### 2.1 Materials, Reagents, and Solvents

Potassium permanganate (≥99%), citric acid monohydrate (99.5–102%), 1-amino-2-propanol (MIPA, 93%), poly(vinyl alcohol) (PVA, 99+% hydrolyzed), and potassium bromide (≥99%, FT-IR grade) were purchased from Sigma-Aldrich. Sodium cholate hydrate (SCH, 99%) was purchased from Alfa Aesar. Orthophosphoric acid (85.35%, analytical grade) and absolute ethanol (≥99.99%) were acquired from Fisher Chemical. The commercial MWCNTs with the reference Nanocyl NC7000 (industrial grade, carbon purity >95%) were supplied by Nanocyl S.A. The textile fabric, 100% woven cotton with a Taffeta construction (warp: 30 yarns, weft: 26 picks), 19.7 tex linear mass, and 110.6 g m^–2^ areal density, was supplied by a local store. Ultrapure water (Millipore, specific resistivity: 18.2 MΩ cm) was used throughout the experiments.

### 2.2 Doping of CNTs with Nitrogen

Commercial CNTs were doped with nitrogen-based groups by ball milling using melamine as the nitrogen precursor in a CNT/melamine mass ratio of 3:1.^21^ Briefly, a mixture of CNTs and melamine was stirred in a Retsch MM 200 horizontal ball mill, at a frequency of 15 s^–1^ for 4 h. The resulting sample was thermally treated at 600 °C for 3 h under controlled nitrogen atmosphere at a flow rate of 100 mL min^–1^. The resulting N-doped material, named CNT-N, contained 1.9 mmol of nitrogen per gram of material (determined by elemental analysis).

### 2.3 One-Pot Fabrication of CNT-N@MO Hybrid Nanomaterials

Two different hybrid nanomaterials consisting of CNT-N functionalized *in situ* with MO NPs (denoted as CNT-N@MO, where MO = MnO_2_ or Mn_3_O_4_) were prepared. *In situ* functionalization of the CNT-N support with the MO nanomaterials was performed in the presence of citric acid (CA), envisaging the controlled growth of the MOs over the CNT-N support.

#### 2.3.1 Synthesis of CNT-N@MnO_2_ Hybrid Nanomaterial

First, 0.350 g of CNT-N were dispersed in 100 mL of an aqueous solution containing 3.0 M MIPA and 0.12 M CA (95:5 V/V) in an ultrasonic bath for 30 min. Subsequently, 20 mL of an aqueous solution of 0.03 M KMnO_4_ were added to the CNT-N dispersion dropwise, and the resulting mixture was magnetically stirred at room temperature for 2 h. After that, the resulting material was separated by centrifugation and submitted to several cycles of washing with Millipore water/centrifugation until a neutral pH was obtained. Finally, the hybrid nanomaterial was washed once with ethanol and dried under vacuum at room temperature overnight. The nanomaterial was denoted as CNT-N@MnO_2__CA.

#### 2.3.2 Fabrication of CNT-N@Mn_3_O_4_ Hybrid Nanomaterial

For the preparation of the CNT-N@Mn_3_O_4_ hybrid nanomaterial, 0.350 g of CNT-N were dispersed in 100 mL of an aqueous solution of 3.0 M MIPA and 0.12 M CA (95:5 V/V) under sonication for 30 min. Subsequently, the resulting dispersion was heated until 100 °C under magnetic stirring, followed by the dropwise addition of 20 mL of 0.03 M KMnO_4_ aqueous solution. The reaction medium was maintained under stirring at that temperature for 24 h. After that, cooling was performed until room temperature and the resulting nanomaterial was centrifuged and washed with Millipore water several times until neutral pH. The final hybrid nanomaterial was washed once with absolute ethanol and dried under vacuum at room temperature overnight. The nanomaterial was denoted as CNT-N@Mn_3_O_4__CA.

MO nanomaterials were also prepared *ex situ* in the absence of the CNT-N support following similar routes to allow the morphological, structural, and chemical comparison with the grafted MO NPs within the hybrids.

### 2.4 Preparation of Textile Electrodes

Cotton fabrics (7.0 × 7.0 cm^2^) were sequential washed with toluene, ethanol, and Millipore water, 15 min each, followed by a drying period of 24 h at room temperature.

Three aqueous CNT-N-based inks were prepared following the procedure reported by Costa et al.^22^ through the dispersion of CNT-N, CNT-N@MnO_2__CA, or CNT-N@Mn_3_O_4__CA into a SCH aqueous solution (10 mg mL^–1^) under sonication for a period of 80 min.

The textile-based electrodes were fabricated through the impregnation of the prewashed cotton fabrics (3.5 × 3.5 cm^2^) with the CNT-N-based dispersions (CNT-N or hybrids) via the dip-pad-dry process. First, the cotton substrate was dipped into the CNT-N-based dispersion and then submitted to a padding process for the removal of the excess of nanomaterial. Afterward, the resulting coated fabric was dried at 100 °C for 10 min. In order to increase the nanomaterial loading and improve the electrical conductivity of the fabric, the dip-pad-dry process was repeated several times until the electrical resistance stabilized.

Finally, each coated cotton fabric (3.5 × 3.5 cm^2^) was cut in two sections, with 3.5 × 1.0 cm^2^ each, that were used as textile electrodes.

### 2.5 Fabrication of Textile Supercapacitors

The PVA/H_3_PO_4_ polymer gel electrolyte was prepared as reported in a previous work.^22^ The solid-gel electrolyte was deposited in one of the faces of each textile electrode in an active area of 2.5 × 1.0 cm^2^, followed by partial air-drying. Then, the textile-based devices were fabricated with a sandwich-type configuration through the assembly of both textile electrodes with the electrolyte in between, followed by complete air-drying. The asymmetric textile SCs containing one textile electrode based on CNT-N and the other based on a hybrid nanomaterial were labeled as CNT-N//CNT-N@MO (MO = MnO_2__CA or Mn_3_O_4__CA). For comparison, symmetric CNT-N//CNT-N and CNT-N@MO//CNT-N@MO textile SCs were also fabricated.

### 2.6 Physicochemical Characterization

X-ray diffraction (XRD) measurements were performed at room temperature on a SmartLab Rigaku diffractometer operated at 9 kW power (40 kV and 200 mA), with Cu Kα radiation (*λ* = 1.5406 Å) and a high-resolution *θ*/*θ* closed loop goniometer drive system, and then numerically converted to a Bragg–Brentano *θ*/2*θ* configuration in the 2*θ* range of 10–90°, at a scan rate of 10° min^–1^, and a step of 0.02°.

Scanning electron microscopy coupled with energy-dispersive X-ray spectroscopy (SEM-EDS) were used for the characterization of all samples. These techniques were performed at the Materials Center of the University of Porto (CEMUP), Portugal, on a high-resolution environmental scanning electron microscope FEI Quanta 400FEG ESEM equipped with an energy-dispersive X-ray spectrophotometer (EDAX Genesis X4M). The original fabric and the MO nanomaterials were coated with Au/Pd by sputtering for 70 s with a current intensity of 15 mA using the SPI-Module Sputter Coater equipment. The CNT-N-based materials and the respective coated fabrics did not require any previous electrically conductive coating.

The transmission electron microscopy (TEM) characterization of the nanomaterials was performed on a JEOL JEM 1400-series microscope operating at an accelerating voltage of 120 kV and coupled with a digital charge coupled device (CCD) camera Orious (1100 W), at the Histology and Electron Microscopy Service (HEMS)/i3S of the University of Porto, Portugal. The samples were prepared through sonication in ethanol, followed by immersion of copper grids coated with a holey carbon film on the resulting dispersions. Subsequently, the grids were left to dry at room temperature.

The Raman spectra of the CNT-N-based nanomaterials were acquired at room temperature on a Jobin–Yvon LabRaman spectrometer equipped with a CCD camera using a He–Ne laser (*λ* = 632.8 nm) with a power of 20 mW. An optical microscope Olympus with an objective lens of 50× was used to focus the laser beam on the samples, as well as to observe the quality of the analyzed areas before and after the measurements. The laser power was reduced 50% by a filter of natural density to avoid the thermal decomposition of the samples. The Raman spectra of the electrodes section of the assembled devices after the cycling tests were acquired at room temperature on a InVia Qontor confocal Raman spectrometer assembled with a Leica DM2700 microscope, using an incident Cobolt 04-01 Series Samba laser of *λ* = 532 nm. For comparison, the Raman spectra of the pristine cotton fabric, CNT-N-based nanomaterials, and textile electrodes before the assembly were acquired using the same equipment.

The nitrogen and manganese contents of the hybrid nanomaterials were determined by elemental analysis and inductively coupled plasma-optical emission spectroscopy, respectively, at Laboratório de Análises, Instituto Superior Técnico, University of Lisbon, Portugal.

The N_2_ adsorption–desorption isotherms of the CNT-based materials were performed at Laboratório de Análises REQUIMTE/LAQV, NOVA School of Science and Technology, NOVA University Lisbon, Portugal, at −196 °C in a gas porosimeter Micromeritics ASAP 2010 (accelerated surface area and porosimetry system). The samples were previously degassed at 150 °C for 3 h under vacuum.

The X-ray photoelectron spectroscopy (XPS) characterization of CNT-N, MnO_2__CA, Mn_3_O_4__CA, and the respective hybrids was performed at CEMUP, on a Kratos AXIS Ultra HAS spectrometer equipped with a monochromatic Al Kα X-ray source (1486.7 eV), operating at 15 kV (90 W), in the Fixed Analyzer Transmission (FAT) mode. All binding energies were calibrated using the C 1s band at 284.6 eV as an internal reference. The XPS spectra were deconvoluted using a nonlinear least-squares fitting routine after a Shirley-type background subtraction using the CasaXPS software (version 2.3.19). The C 1s band of the component assigned to sp^2^ C=C of CNT-N-based nanomaterials was fitted using the Lorentzian Finite (LF) asymmetric line shape with an overall asymmetry index of 0.14 based on a previous report.^23^ The remaining C 1s components and the other core-level regions were fitted using a symmetrical Gaussian–Lorentzian function. The spectra of Mn 2p_3/2_ were resolved according to the fitting procedures and parameter constrains defined by Biesinger et al. and Ilton et al.,^24,25^ which take into account the multiplet splitting of the various oxidation states of manganese cations and overlapping of the corresponding deconvoluted bands occurring in the same binding energy range.

### 2.7 Electrochemical Evaluation of Textile SCs

The electrochemical performance of the textile SCs was evaluated at room temperature by electrochemical impedance spectroscopy (EIS), cyclic voltammetry (CV), and galvanostatic charge/discharge experiments (GCD) in a standard two-electrode cell configuration, using an AutoLab PGSTAT 20 potentiostat (EcoChimie). The EIS measurements were performed in the frequency range of 0.1 Hz–1.0 MHz with 0 V of potential and an AC amplitude of 25 mV. The fitting of the EIS data was performed using NOVA 2.1 software. The *i*–*V* curves were acquired over the potential window of −1.0 to 1.0 V, at scan rates of 1, 5, 10, 25, 50, 75, and 100 mV s^–1^. GCD experiments were performed at current density values of 0.10, 0.15, and 0.20 mA cm^–2^. Cycling performance tests were conducted by CV at 10 mV s^–1^ for 8000 charge/discharge cycles.

The specific capacitance (*C*_T_) of the devices was determined through the respective *i*–*V* cycles using the following equation^26^
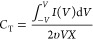
1where *I* is the current intensity (in A), υ is the potential scan rate (V s^–1^), Δ*V* is the applied potential window (in V), and *X* is the total mass of active material, area, or volume of the active region of the device (expressed in g, cm^2^, or cm^3^, respectively).

The energy density (*E*) of the device per unit of active mass, area, or volume (in W h kg^–1^, W h cm^–2^, or W h cm^–3^, respectively) was determined through the following equation^26^
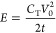
2where *C*_T_ is the specific capacitance of the device (in F kg^–1^, F cm^–2^ or F cm^–3^), *V*_0_ is the operation potential (in V), and *t* is a conversion factor of time (3600 s).

The power density (*P*) of the device per unit of active mass, area, or volume (in W kg^–1^, W cm^–2^ or W cm^–3^) was calculated by the following equation^26^
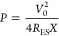
3where *R*_ES_ corresponds to the equivalent series resistance of the device (in Ω) determined by EIS, and *X* is the total mass of active material, area, or volume of the active region of the device (expressed in kg, cm^2^, or cm^3^).

## 3 Results and Discussion

### 3.1 Morphological and Structural Characterization of Mn_*x*_O_*y*_-Based Nanomaterials

**Figure 1:**
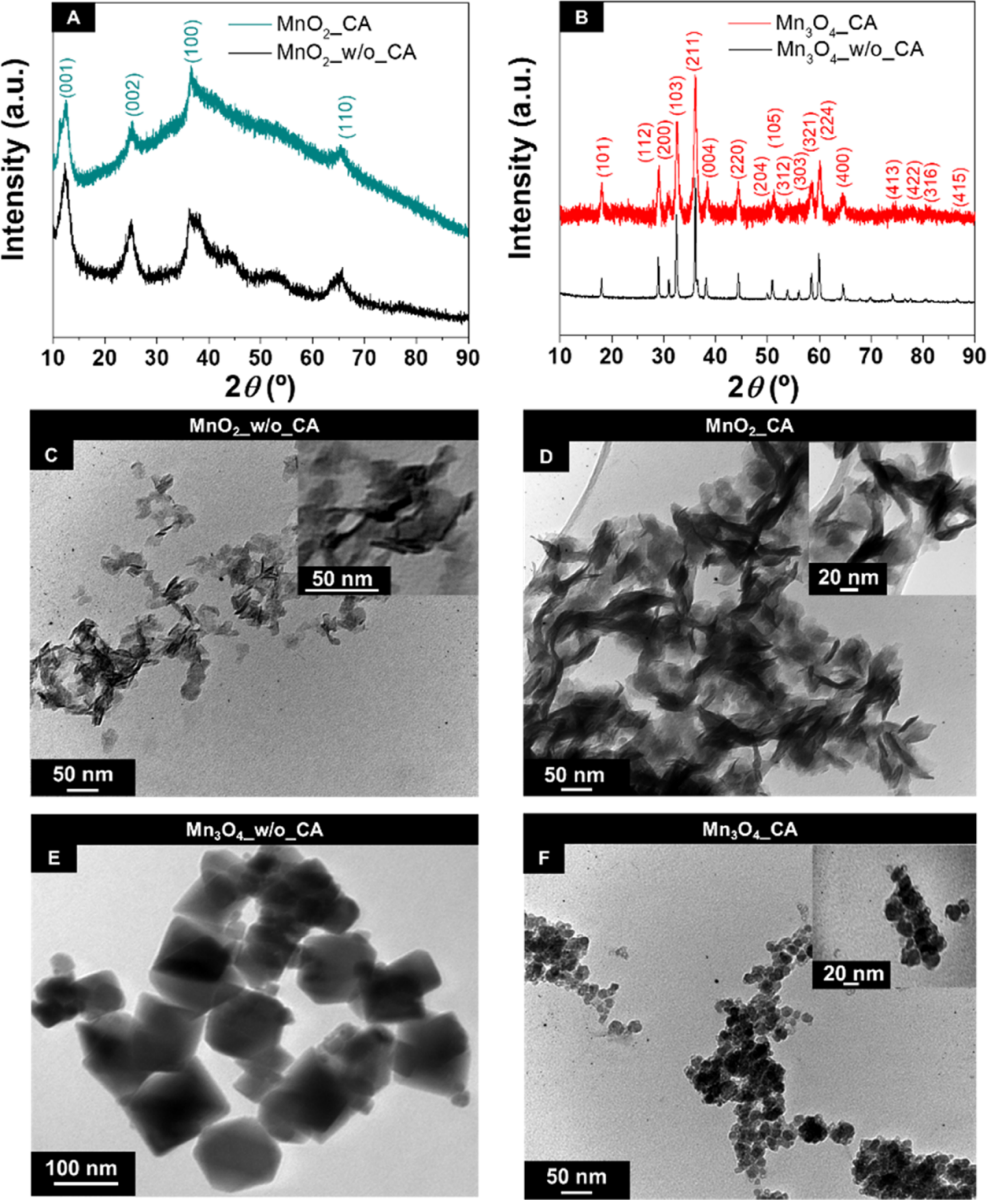
(A, B) X-ray diffractograms and (C–F) TEM images at 200 000× (C, D, and F) and 130 000× magnifications (E) of MnO_2_ and Mn_3_O_4_ nanomaterials prepared in the absence and presence of CA. Inset images in panels (C, D, and F): magnified TEM images with 300 000× (C), 600 000× (D), and 500 000× magnification (F).

Prior to the preparation of the CNT-N@MO hybrids, MO nanomaterials were synthesized *ex situ* in the absence of the CNT-N support and optimized in terms of the structure, type of phase, and morphology by changing several experimental parameters, including the reaction temperature, the reaction time, and the presence/absence of a chelating agent (*i.e*., citric acid). The variation of the temperature of the reaction from room temperature to 100 °C resulted in the modification of the MO phase from birnessite (MnO_2_) to hausmannite (Mn_3_O_4_), as unveiled by XRD (Figure 1A vs. Figure 1B).

The XRD patterns of MnO_2_ prepared in the absence and presence of CA (samples denoted as MnO_2__w/o_CA and MnO_2__CA, respectively) display peaks at 2*θ* = 12.4, 25.4, 36.9, and 65.8°, which are assigned to the crystallographic planes (001), (002), (100), and (110), respectively, of the hexagonal birnessite phase of manganese(IV) dioxide with space group *P*6_3_/*mmc* (Figure 1A).^27^ No additional phases are detected in both diffractograms. The X-ray pattern of the MnO_2_ NPs prepared in the presence of CA is less defined than that of the NPs prepared in the absence of CA, revealing the lower crystallinity of the material.

The diffractograms of Mn_3_O_4_ prepared without or with CA (Mn_3_O_4__w/o_CA and Mn_3_O_4__CA, respectively) present peaks at 2*θ* = 18.1, 28.9, 30.9, 32.5, 36.0, 38.1, 44.4, 50.1, 50.9, 53.8, 56.2, 58.5, 59.8, 64.5, 74.2, 76.6, 80.6, and 86.6°, which match the (101), (112), (200), (103), (211), (004), (220), (204), (105), (312), (303), (321), (224), (400), (413), (422), (316), and (415) reflections, respectively (Figure 1B), of spinel-type tetragonal hausmannite with the *I*4_1_/*amd* spatial group.^27^ As previously observed for the MnO_2_ material, the addition of CA during the synthesis of Mn_3_O_4_ also led to broader diffraction peaks in the corresponding diffractogram. The X-ray patterns of both Mn_3_O_4_-based samples do not present additional peaks, demonstrating the high purity of the samples without secondary phases. Remarkably, these pure hausmannite materials were obtained through the same one-step precipitation route used to synthesize MnO_2_, by only increasing the reaction temperature from room temperature to 100 °C. In addition to the mild reaction conditions, no posterior thermal treatment step was required for the fabrication of Mn_3_O_4_, in opposition to several multistep synthesis routes reported in the literature, which required reaction temperatures above 150 °C and/or thermal annealing.^28,29^

TEM and SEM characterization of the MnO_2_-based nanomaterials synthesized at room temperature (Figures 1C,D and S1A) reveals that they present identical nanosheet-like morphology, regardless of the absence or presence of CA during their fabrication.^30^ In the case of Mn_3_O_4_-based samples, the addition of CA during the nanomaterial fabrication at a higher temperature (100 °C) induces a change of the morphology from cubic to spherical (Figures 1E,F and S1B) and a major reduction of the particles' dimensions, leading to an average particle size of ∼17 ± 5 nm (by TEM). Thus, both TEM and SEM techniques corroborate the XRD results, highlighting the importance of combining a higher reaction temperature with CA in the tuning of the morphology and particle size of the Mn_3_O_4_ nanomaterial due to its chelating properties.^31^ In fact, CA can coordinate to the metal cations on the surface of the material, leading to heterogeneous MO nucleation. Additionally, CA can adsorb to the surface of the as-formed MO particles, restraining their growth and leading to a smaller particle size.^32^

Considering the abovementioned results, CNT-N@MO hybrid nanomaterials were synthesized in the presence of a CA chelating agent for the controlled growth of the MO nanoparticles over the CNT-N support. Initially, the CNTs were doped with nitrogen (CNT-N) in order to create active sites for the immobilization of the MO NPs. Afterward, two hybrid CNT-N@MO nanomaterials were prepared by *in situ* immobilization of the MO NPs on the surface of the CNT-N material through a one-pot precipitation process in the presence of CA. For each hybrid nanomaterial, the CNT-N/metal salt molar ratio used during the synthesis was selected taking into account the total amount of nitrogen on the CNT-N support (determined by elemental analysis).

**Figure 2:**
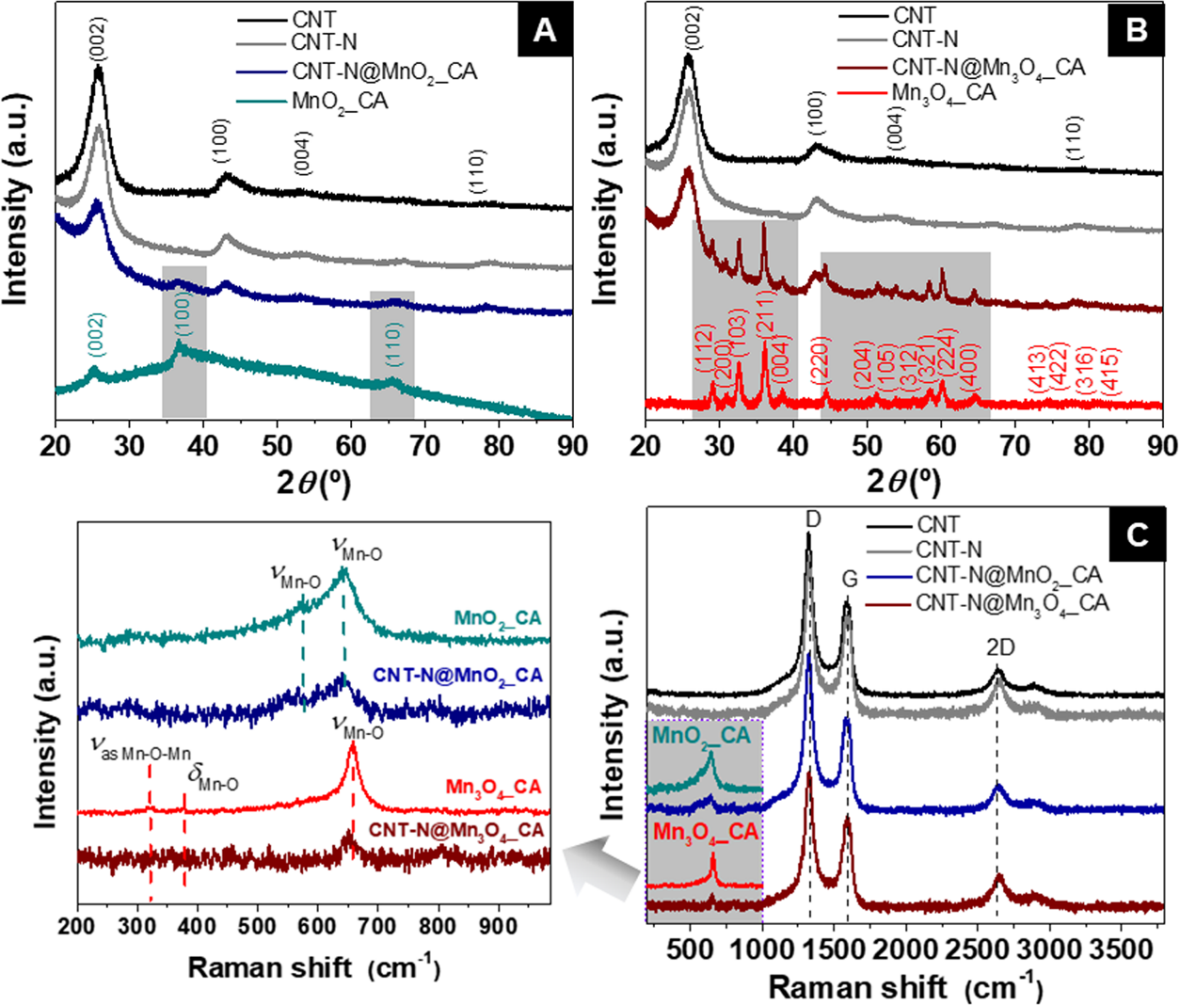
(A, B) X-ray patterns and (C) Raman spectra of CNT, CNT-N, CNT-N@MnO_2__CA, and CNT-N@Mn_3_O_4__CA in the range of 200–3800 cm^–1^ and acquired with a *λ* = 632.8 nm laser. Gray rectangles in panels (A) and (B): diffraction peaks corresponding to the Bragg reflections of the MO nanomaterials within the hybrids. Left side of panel (C): magnification of the Raman spectra in the region of the bands assigned to the characteristic MO vibration modes.

The diffractogram of the pristine CNT sample (Figure 2A) exhibits peaks at 2*θ* = 25.8, 42.9, 53.1, and ∼78.3°, corresponding to the (002), (100), (004), and (110) planes, characteristic of a graphitic structure.^33^ The X-ray pattern of CNT-N is similar to that of the pristine CNT, proving that nitrogen doping through ball milling did not damage the CNT structure. The interlayer spacing values (*d_hkl_*, where *h*, *k*, and *l* indexes are the Miller indexes that define the orientation of different atomic planes) for both CNT and CNT-N materials were obtained through the (002) main reflection of the corresponding diffractograms using Bragg’s law^34^
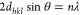
4where *θ* is the incident angle (angle between the scattered plane and the incident X-ray beam), *n* is the reflection number (integer value), and *λ* is the wavelength of the incident radiation.

The *d_hkl_* values of both materials are similar (3.446 Å for CNT-N vs. 3.457 Å for CNT), suggesting the preservation of the interwall distance within the CNT structure upon nitrogen doping.

In the case of the X-ray patterns of the hybrid nanomaterials (Figure 2A,B), the characteristic reflections associated with both the grafted MO NPs (highlighted in gray rectangles) and CNT-N can be detected, confirming the successful immobilization of the MOs onto the CNT-N structure, as well as the preservation of both the type of MO phase and CNT-N integrity. Moreover, the relative intensity of the (002) reflection associated with the CNT-N support is higher than that of the strongest reflection assigned to the grafted MO NPs ((100) and (211) for MnO_2__CA and Mn_3_O_4__CA, respectively), indicating that CNT-N is acting as a supporting matrix.

Interestingly, pure hausmanite phase was obtained in the case of CNT-N@Mn_3_O_4_ at 100 °C, without requiring any *post*-thermal treatment step, which contrasts with typical synthetic routes reported in the literature to yield the hausmannite phase in carbon-based hybrid materials, including hydrothermal/solvothermal (160–180 °C) and coprecipitation methods.^35−37^

The average crystallite size (*D*_XRD_) of the Mn_3_O_4_ NPs prepared *ex situ* and incorporated in the hybrids was calculated by the Debye–Scherrer equation^38^
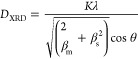
5where *K* is a dimensionless factor (*K* = 0.9 for spherical particles), λ is the wavelength of the X-ray source, *θ* and *β*_m_ are the diffraction angle and full width at half-maximum, respectively, of the most intense reflection assigned to the MO NPs (*i.e*., (211) for Mn_3_O_4__CA), and *β*_s_ corresponds to the instrumental broadening (considered negligible).

The crystallite size of MnO_2__CA-based nanomaterials was not calculated considering their complex morphology (nonspherical flake-like), leading to peak broadening in the corresponding XRD patterns. In the case of the Mn_3_O_4__CA NPs grafted to the CNT-N support, the *D*_XRD_ value is comparable to that of the corresponding MO prepared *ex situ* (18.6 vs. 19.8 nm, respectively).

**Figure 3:**
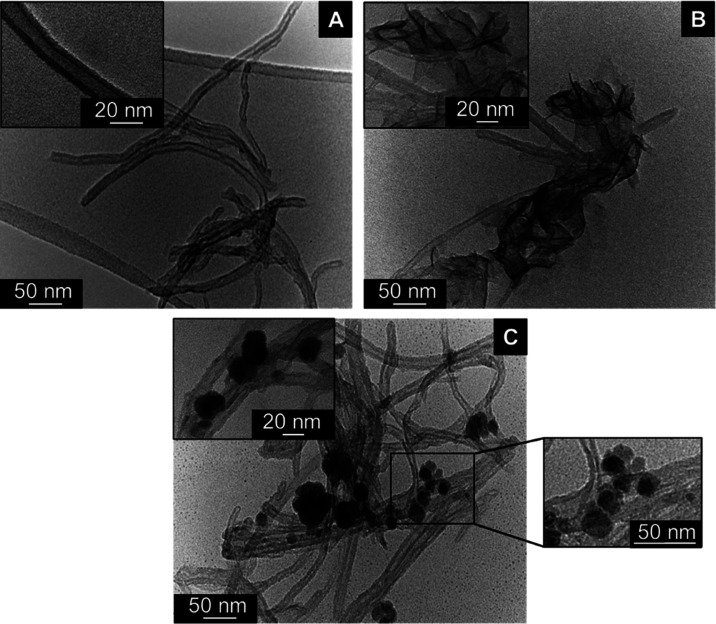
TEM micrographs of (A) CNT-N, (B) CNT-N@MnO_2__CA, and (C) CNT-N@Mn_3_O_4__CA at 300 000× and 500 000× (insets) magnifications.

TEM micrographs of CNT-N (Figure 3A) reveal a tubular-like morphology characteristic of CNTs,^22^ with the nanotubes presenting average outer and inner diameters of 13.4 ± 3.3 and 5.2 ± 2.4 nm, respectively, with a wall thickness of 8.2 ± 0.8 nm. The CNT-N has similar dimensions to those of the pristine CNTs (average outer and inner diameters of 14.3 ± 1.2 and 3.9 ± 0.7 nm, respectively; Figure S2), but with a reduction of the length of the nanotubes as a result of the ball-milling step.^39^ Nevertheless, the carbon material structure was preserved upon N-doping, corroborating the XRD results.

TEM images and EDS characterization of CNT-N@MnO_2__CA and CNT-N@Mn_3_O_4__CA hybrids (Figure 3B,C and Figure S3) confirm the efficacious grafting of MnO_2__CA and Mn_3_O_4__CA NPs throughout the CNT-N support, with the morphology of the NPs being maintained when compared to that of the pure MO counterparts prepared *ex situ* (Figure 1D,F). Moreover, the CNT-N supporting material preserved its tubular-like structure upon immobilization of the MO NPs, in accordance with XRD results.

In the case of CNT-N@MnO_2__CA material, the grafted MnO_2_ NPs have a wrinkled nanoflake morphology (Figure 3B). On the other hand, the TEM images of CNT-N@Mn_3_O_4__CA (Figure 3C) reveal the presence of single quasi-spherical Mn_3_O_4_ NPs with an average size of ∼16 ± 4 nm (similar to the dimensions of the MO NPs prepared *ex situ*, ∼17 ± 5 nm) and a few clusters of particles with ∼32–57 nm of diameter.

Finally, no free MO NPs are observed in the TEM images of the hybrids, revealing that the one-pot precipitation route and the selection of N-doped CNTs as supporting matrix was an excellent strategy to ensure the selective grafting of the MO NPs onto the CNT-N surface during their formation, and prevent the nucleation/growth of free MO NPs.^40^

The Raman spectra of both pristine and N-doped CNT, acquired with a *λ* = 632.8 nm laser and presented in Figure 2C, exhibit three bands characteristic of graphitic materials: D, G, and 2D bands at 1322–1326, 1589–1593, and 2640–2648 cm^–1^, respectively (Table 1). The D and G bands are assigned to out-of-plane and in-plane vibrations from A_1g_ and E_2g_ modes, respectively. More specifically, in sp^2^-type carbon-based materials, the D band arises from the presence of disorder in the graphitic structure, while the G band is associated with the tangential C–C stretching vibrations of the graphitic structure, describing the level of order.^41^ The 2D band is assigned to the double resonant contribution of the same phonon (A_1g_ forward and backward scattering), being attributed to the second-order overtone of the D band.^41^ The *I*_D_/*I*_G_ ratio, which quantifies the degree of structural disorder of graphitic structures arising from the presence of defects, such as vacancies, heteroatoms, and/or impurities,^41^ was calculated for all materials (Table 1).

**Table 1:** Raman Shifts, *I*_D_/*I*_G_ Ratios, and Textural Properties of CNT, CNT-N, CNT-N@MnO_2__CA, and CNT-N@Mn_3_O_4__CA Nanomaterials

	Raman shift (cm^–1^)					
sample	band D	band G	band 2D	*I*_D_/*I*_G_^a^	*S*_BET_^b^ (m^2^ g^–1^)	*V*_p_^c^ (cm^3^ g^–1^)
CNT	1322	1589	2640	1.78	226	0.423
CNT-N	1326	1593	2648	1.68	253	0.547
CNT-N@MnO_2__CA	1323	1588	2643	1.82	256	0.567
CNT-N@Mn_3_O_4__CA	1324	1590	2645	1.96	219	0.527

a *I*_D_/*I*_G_ is the ratio between the intensity of the D and G bands assigned to CNT-N in the Raman spectra of the materials.

b Specific surface area.

c Total pore volume determined at *P*/*P*_0_ = 0.95.

The D, G, and 2D bands in the Raman spectrum of CNT-N are slightly shifted toward higher wavenumbers relative to those in the spectrum of the pristine CNT (Table 1: shifts of 4, 4, and 8 cm^–1^, respectively), suggesting the existence of chemical modifications within the CNT-N framework induced by the doping of the CNTs with nitrogen-based groups.^42^ The *I*_D_/*I*_G_ ratio slightly decreases ongoing from CNT to CNT-N (from 1.78 to 1.68), suggesting that the carbon nanotubes structure was preserved upon the doping process, *i.e*., after the ball-milling step and subsequent thermal treatment (at 600 °C).^43^

In the spectra of both hybrid materials, in addition to the characteristic bands from CNT-N (Figure 2C), additional low-intensity bands are observed in the range of 200–750 cm^–1^, which are assigned to the grafted MO NPs (Mn–O groups). In particular, the Raman spectrum of CNT-N@MnO_2__CA exhibits two bands at 568 and 647 cm^–1^ (left side of Figure 2C), which are assigned to Mn–O symmetric stretching vibrations in the basal plane of [MnO_6_] sheets and Mn–O stretching vibrations of [MnO_6_] sheets, respectively, characteristic of birnessite.^44^ The Raman spectrum of CNT-N@Mn_3_O_4__CA presents an intense band at 657 cm^–1^ assigned to the A_1g_ mode of Mn–O breathing vibrations of Mn^2+^ in tetrahedral coordination, characteristic of the Mn_3_O_4_ spinel structure.^45,46^ The low-intensity vibrational bands of Mn_3_O_4_ at 320 and 375 cm^–1^, assigned to asymmetric stretching of bridge oxygen species in Mn–O–Mn (E_g_ mode) and bending vibrations of Mn–O (T_2g_ mode), respectively,^47^ were not observed in the Raman spectrum of the hybrid Mn_3_O_4_-based material, suggesting a lower loading of grafted Mn_3_O_4_.

The D, G, and 2D bands assigned to the CNT-N support in the Raman spectra of both CNT-N@MnO_2__CA and CNT-N@Mn_3_O_4__CA present a shift toward lower wavenumbers when compared to the Raman spectrum of CNT-N (CNT-N@MnO_2__CA: 3, 5, and 5 cm^–1^, respectively; CNT-N@Mn_3_O_4__CA: 2, 3, and 3 cm^–1^, respectively), suggesting the existence of interactions between CNT-N and MO. The *I*_D_/*I*_G_ ratios for the hybrid materials are higher than that of CNT-N (1.82 and 1.96 for CNT-N@MnO_2__CA and CNT-N@Mn_3_O_4__CA, respectively, vs. 1.68 for CNT-N), owing to the chemical modification of the CNT-N support upon grafting of the MOs.

The textural properties of the CNT-based materials were characterized by N_2_ sorption studies at −196 °C. The N_2_ adsorption–desorption isotherms of all samples are of type II with H3-type hysteresis loops (Figure S4) according to IUPAC classification.^48^ Upon doping of the CNT nanomaterial with nitrogen by ball milling, its specific surface area (*S*_BET_) increased from 226 to 253 m^2^ g^–1^ and the pore volume (*V*_p_) increased from 0.423 to 0.547 cm^2^ g^–1^ (Table 1, CNT vs. CNT-N), in accordance with the literature.^19^

The *S*_BET_ and *V*_p_ values of CNT-N and CNT-N@MnO_2_, presented in Table 1, are comparable (253 vs. 256 m^2^ g^–1^ and 0.547 vs. 0.567 cm^–3^ g^–1^), while CNT-N@Mn_3_O_4_ presents slightly lower *S*_BET_ (219 m^2^ g^–1^) and *V*_p_ (0.527 cm^3^ g^–1^). The differences in the specific surface area and pore volume of both hybrids can be probably attributed to the distinct morphology of the immobilized MO NPs. In particular, the more open and wrinkled-like morphology of the grafted MnO_2_ particles within the CNT-N@MnO_2_ hybrid (in contrast to the immobilized spherical-like Mn_3_O_4_ NPs) may play a key role in the preservation of the textural properties.

### 3.2 Chemical Characterization of Hybrid CNT-N-Based Nanomaterials

**Figure 4:**
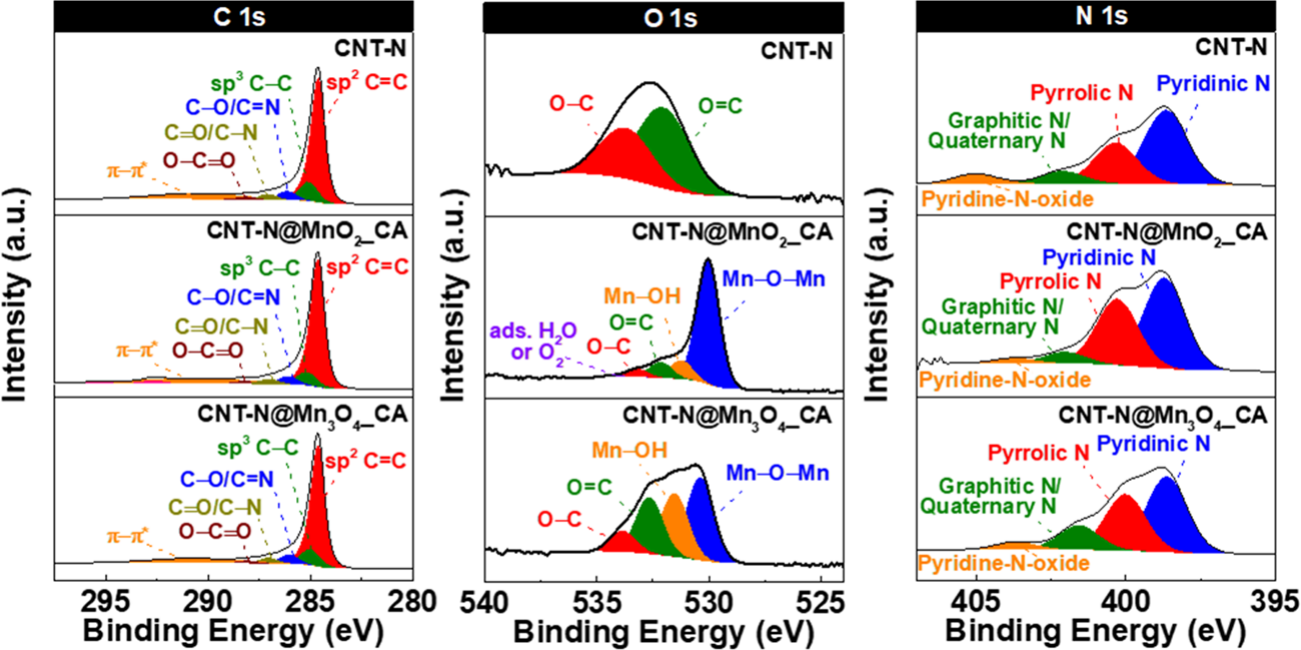
Deconvoluted C 1s, O 1s, and N 1s high-resolution XPS spectra of CNT-N, CNT-N@MnO_2__CA, and CNT-N@Mn_3_O_4__CA.

**Figure 5:**
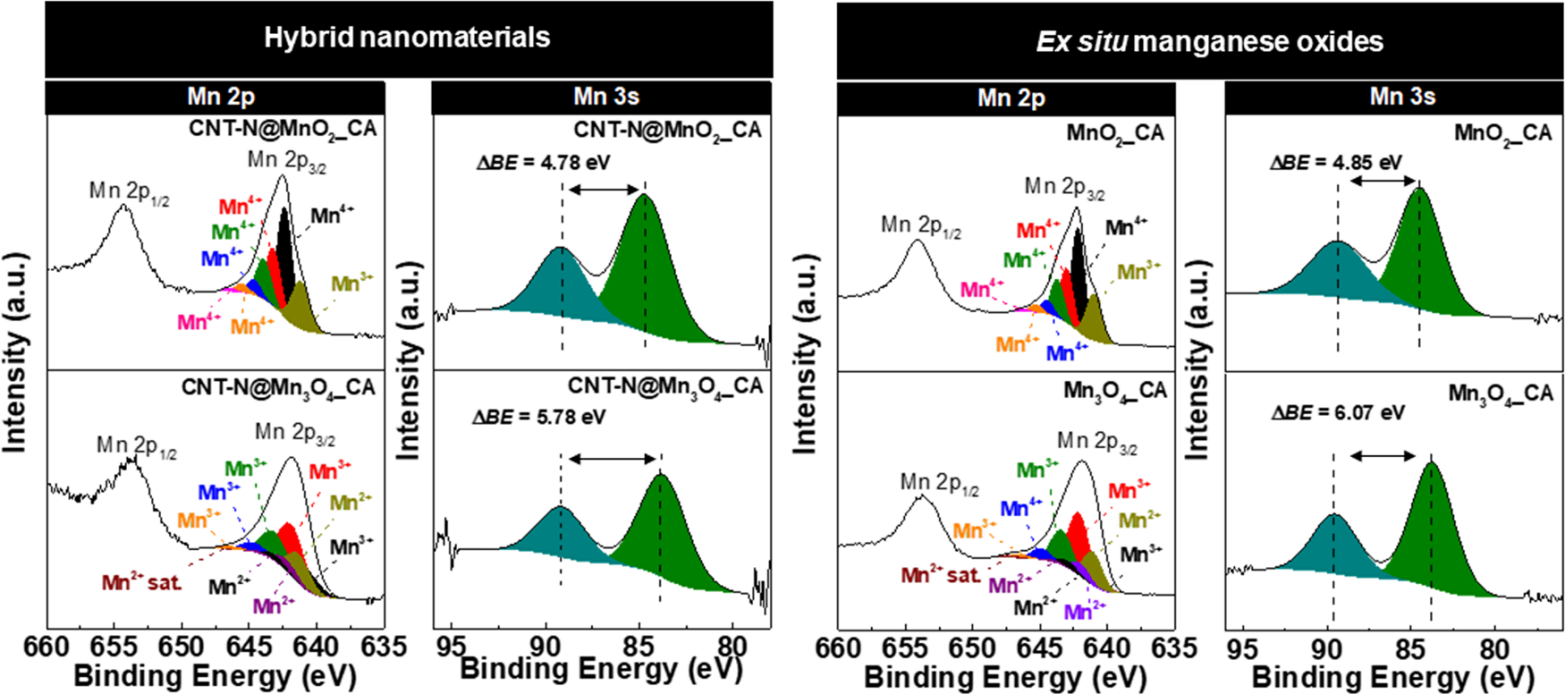
Mn 2p and Mn 3s high-resolution spectra of the hybrids and *ex situ* MO-based nanomaterials.

The as-prepared nanomaterials were characterized by XPS in order to obtain information about the atomic percentages and binding energies (BEs) of the different elements present on their surfaces (Figures 4 and 5, and Tables 2, S1 and S2). The CNT-N material mainly contains carbon (95.0 atom %), nitrogen (2.2 atom %), and oxygen (1.9 atom %) on its surface (Table 2), confirming its doping with nitrogen-based groups and the presence of oxygen-based functionalities. A slight amount of aluminum was detected, which can be attributed to residues arising from the industrial process used in the preparation of the CNTs. The nitrogen surface content determined by XPS is comparable to the bulk loading obtained by elemental analysis (1.8 vs. 1.9 mmol g^–1^, Table 2), suggesting that the nitrogen-based groups are homogeneously distributed throughout the nanotubes structure.

**Table 2:** Surface Atomic Percentages for CNT-N-Based Nanomaterials Obtained by XPS and the Amount of N and Mn Determined by XPS and Chemical Analysis

								chemical analysis (mmol g_material_^–1^)			
	atomic % (by XPS)^a^							N		Mn	
nanomaterial	C 1s	O 1s	N 1s	Mn 2p	Al 2p	Si 2p	K 2p	XPS	EA^b^	XPS	ICP^b^
CNT-N	95.0	1.9	2.2	–	0.9	–	–	1.8	1.9	–	–
CNT-N@MnO_2__CA	86.2	7.6	2.3	3.5	–	–	0.4	1.6	1.7	2.5	1.1
CNT-N@Mn_3_O_4__CA	89.0	6.1	2.2	1.2	0.8	0.7	–	1.7	1.1	0.9	0.7

a Determined by the areas of the respective deconvoluted bands in the high-resolution XPS spectra.

b EA—elemental analysis; ICP-OES—inductively coupled plasma-optical emission spectroscopy.

The C 1s high-resolution spectrum of CNT-N (Figure 4) presents an asymmetric line shape and was deconvoluted in six bands at 284.6, 285.1, 286.1, 287.1, 288.1, and 290.6 eV, associated with sp^2^ C=C, sp^3^ C–C, C–O, and/or C=N, >C=O, and/or C–N, O–C=O and π–π* satellite band of the sp^2^-hybridized carbon, respectively (Table S1).^49^

The O 1s high-resolution spectrum of CNT-N exhibits two bands at 532.0 and 533.7 eV, assigned to O=C and O–C bonds, respectively.^50^ The presence of both bands corroborates the existence of oxygen-containing functionalities in the CNT-N support.

The N 1s high-resolution spectrum of CNT-N is resolved into four bands located at 398.6, 400.2, 401.7, and 404.2 eV, which are attributed to pyridinic-N (54.8%), pyrrolic-N (30.5%), graphitic/quaternary N (10.1%), and pyridine-N-oxide groups (4.6%), respectively, revealing the presence of four types of nitrogen-based functionalities on the surface of the support.^51^ The prevailing groups are pyridinic and pyrrolic. These results are in accordance with previous works on N-doping of CNTs through the ball-milling process.^21^

Concerning the hybrid nanomaterials, both contain C, O, N, and Mn on their surface (Table 2). A residual amount of K (0.4 atom %) was also detected in CNT-N@MnO_2__CA, which can be attributed to the metal cation precursor used in the synthesis process (KMnO_4_). CNT-N@MnO_2__CA presents higher Mn and O surface contents than CNT-N@Mn_3_O_4__CA (atom % Mn: 3.5% vs. 1.2%; atom % O: 7.6% vs. 6.1%), indicating the presence of a higher amount of MnO_2_ on the CNT-N surface. The nitrogen surface content of both hybrids is very similar to that obtained for the CNT-N material (2.3 and 2.2 atom % for CNT-N@MnO_2__CA and CNT-N@Mn_3_O_4__CA, respectively vs. 2.2 atom % for CNT-N). On the other hand, for both hybrids, the oxygen surface loading is higher than that of CNT-N (6.1–7.6 atom % vs. 1.9 atom %), while the carbon loading follows the opposite trend (86.2–89.0 atom % vs. 95.0 atom %), confirming the immobilization of the MO on the CNT-N surface. The Mn surface loadings of both hybrids are higher than those obtained by chemical analysis (CNT-N@MnO_2__CA: 2.5 vs. 1.1 mmol g^–1^; CNT-N@Mn_3_O_4__CA: 0.9 vs. 0.7 mmol g^–1^), indicating that the MO NPs are preferentially located on the surface of the carbon support.^52^ These results prove the immobilization of the MO nanomaterials onto the CNT-N surface, as previously attested by XRD, TEM, and Raman spectroscopy, as well as the higher loading of MO in the CNT-N@MnO_2__CA hybrid.

In order to unveil the oxidation states of Mn cations in both the hybrids and corresponding MO nanomaterials prepared *ex situ*, the Mn 2p and Mn 3s high-resolution spectra were deconvoluted (Figure 5). In the case of the Mn 2p spectra, only the Mn 2p_3/2_ region was fitted due to the different shapes of the 2p_3/2_ and 2p_1/2_ bands, probably arising from mixed valence effects in the satellite region, which could lead to misleading results.^24,25^ Thereby, the Mn 2p_3/2_ region was resolved considering the fitting procedures and parameter constrains defined by Biesinger et al. and Ilton et al.,^24,25^ which take into account the multiplet splitting of the oxidation states of manganese cations and overlapping of the corresponding deconvoluted bands occurring in the same binding energy range.

The Mn 2p_3/2_ high-resolution spectral profiles of CNT-N@MnO_2__CA and CNT-N@Mn_3_O_4__CA are similar to those of the respective MO nanomaterials prepared *ex situ* and were deconvoluted in the same number/type of bands. The deconvoluted Mn 2p_3/2_ spectrum of CNT-N@MnO_2__CA (Figure 5) reveals the existence of six bands in the range of 642.4–646.5 eV (642.4, 643.2, 643.9, 644.7, 645.5, and 646.5 eV), which confirms the presence of Mn^4+^ cations (with a total relative area of 80.1%, see Table S1). An additional band at 641.2 eV (with a relative area of 19.9%) is observed, which is assigned to Mn^3+^ cations.^25^ The Mn^4+^/Mn^3+^ ratios for both CNT-N@MnO_2__CA and MnO_2__CA are 4.0, highlighting the predominant oxidation state of +4. Additionally, the splitting between the two bands in the Mn 3s XPS spectra of CNT-N@MnO_2__CA and MnO_2__CA (Figure 5), ΔBE, is 4.75 and 4.85 eV, respectively, confirming that both samples mainly contain Mn^4+^ cations, with some Mn^3+^ contribution. The obtained ΔBE values are characteristic of birnessite.^25^

The Mn 2p_3/2_ spectrum of CNT-N@Mn_3_O_4__CA (Figure 5) reveals the presence of multiple oxidation states assigned to Mn^3+^ (bands at 640.8, 641.9, 643.2, 644.7, and 646.3 eV) and Mn^2+^ cations (bands at 640.9, 641.3, 642.2, 643.2, and 644.3 eV and a satellite peak at 647.6 eV), both characteristics of Mn_3_O_4_.^25^ The relative area of the Mn^3+^ component in both the Mn_3_O_4__CA-based nanomaterials is twice the value of the relative area associated with the Mn^2+^ component (hybrid: 67.4% vs. 32.6%; Mn_3_O_4__CA: 66.9% vs. Mn^2+^: 33.1%), which is close to the ideal stoichiometry for Mn^2+^[Mn^3+^]_2_[O^2–^]_4_.^25^ In the case of the Mn 3s spectra of CNT-N@Mn_3_O_4_ and free Mn_3_O_4_ (Figure 5), the ΔBE values are 5.78 and 6.07 eV, respectively, being in the range of the ΔBEs of Mn^2+^ and Mn^3+^ species (Mn^2+^ = 6.0 eV; Mn^3+^ ≥ 5.3 eV).^53^ These results thus corroborate the identification of the MO phase in this set of nanomaterials as being hausmannite.

The N 1s high-resolution spectra of both hybrids present the four characteristic bands assigned to the N-based functionalities of the CNT-N support (Figure 4 and Table S1). Nevertheless, there is a change in the spectral profile of the first two N 1s components at 398.6 and 400.2 eV, with the relative amount of pyridinic-based groups (398.6 eV) decreasing from 54.8 to 45.9–51.9% ongoing from CNT-N to the hybrids, and the relative amount of the pyrrolic-N functionalities (400.2 eV) increasing from 30.5 to 34.3–38.1%. The increase of the area of the band assigned to pyrrolic-N may be due to the contribution from C–N groups of MIPA alkaline agent, while the decrease of the area of the band ascribed to pyridinic functionalities suggests that the MO NPs are mainly grafted to this type of functional groups on the CNT-N surface. In fact, the pyridinic-N atoms have been reported as metal coordination sites due to their electron-donating properties.^54^ Furthermore, *ab initio* calculations of the geometry/energy of metal–nitrogen sites in CNTs have revealed that the binding between pyridinic-N and transition-metal cations is more energetically favorable than that involving pyrrolic-N sites.^55^ Nevertheless, according to the literature, an additional N 1s component related to nitrogen–metal bonds can be present around ∼399 eV, which falls between the BEs of the bands assigned to pyridinic and pyrrolic-based groups.^56^ Although the XPS software could not resolve that additional contribution from N–Mn(II)/Mn(III)/Mn(IV) bonds, the spectral broadening observed in that range, especially in the N 1s spectrum of CNT-N@Mn_3_O_4__CA, and the decrease in the area of the band related to pyridinic-N functionalities, reinforce the presence of that additional N–metal component and overlapping with the bands related to pyridinic and pyrrolic groups.^40^

Concerning the O 1s high-resolution spectra of the hybrid materials (Figure 4), two bands related to Mn–O–Mn at 530.0–530.5 eV and to Mn–OH at 531.2–531.6 eV are observed, both characteristic of the grafted MOs.^57^ Additional bands are observed at 532.2–532.7 and 533.2–533.9 eV, corresponding to O=C and O–C bonds^58^ from the CNT-N support. A small band at 534.4 eV is also detected in the O 1s spectrum of CNT-N@MnO_2__CA, which is attributed to adsorbed water or molecular oxygen.^57^

The C 1s high-resolution spectra of both hybrid nanomaterials present similar profiles to that of the pristine CNT-N (Figure 4), being deconvoluted into the six characteristic bands of the carbon support, with comparable BE values. These results attest that the structure of the support was preserved upon the incorporation of MO NPs, in accordance with XRD, Raman spectroscopy, and TEM.

### 3.3 Characterization of Textile Electrodes

The as-prepared CNT-N-based samples were used as active electrode nanomaterials for the fabrication of both symmetric and asymmetric textile SCs. For this purpose, textile electrodes were prepared by impregnation of woven cotton fabric substrates in CNT-N-based aqueous dispersions through the dip-pad-dry method, which is a scalable, economically and environmentally viable process used for fabrics dyeing in the textile Industry. During the impregnation process, the amount of nanomaterial incorporated on the cotton substrates (3.5 × 3.5 cm^2^) after each dip-pad-dry step was measured, as well as the electrical resistance until it reached stabilization (Figure S5).

As expected, the incorporation of electrically conductive carbon-based nanomaterials into the natural cotton textile fabric was crucial to reduce its intrinsic nonconductive nature, which is highly important to enhance the performance of SCs. Regardless of the nanomaterial incorporated on the cotton substrate, the normalized resistance of the coated fabrics (electrical resistance of the coated cotton fabric normalized by the mass of incorporated CNT-N-based nanomaterial) decreases almost three orders of magnitude with the increase of the number of incorporation steps (Figure S5A), confirming an improvement of the electrical conductivity. In particular, the cotton fabric coated with CNT-N presents the highest electrical conductivity (specific resistance = 18.5 ± 0.3 Ω cm^–2^ after eight impregnation steps), followed by the fabrics impregnated with CNT-N@Mn_3_O_4__CA (35.8 ± 0.9 Ω cm^–2^ after 16 impregnation steps) and CNT-N@MnO_2__CA (50.2 ± 2.9 Ω cm^–2^ after 11 impregnation steps).

The nanomaterial loading in the coated fabrics (Figure S5B) reached 15.4, 16.7, and 18.7 wt % for cotton_CNT-N, cotton_CNT-N@MnO_2__CA, and cotton_CNT-N@Mn_3_O_4__CA, respectively. Despite the lower nanomaterial loading in cotton_CNT-N, it presents the highest electrical conductivity, while the textiles coated with the hybrids have a slightly more resistive behavior associated with the presence of the MOs.

**Figure 6:**
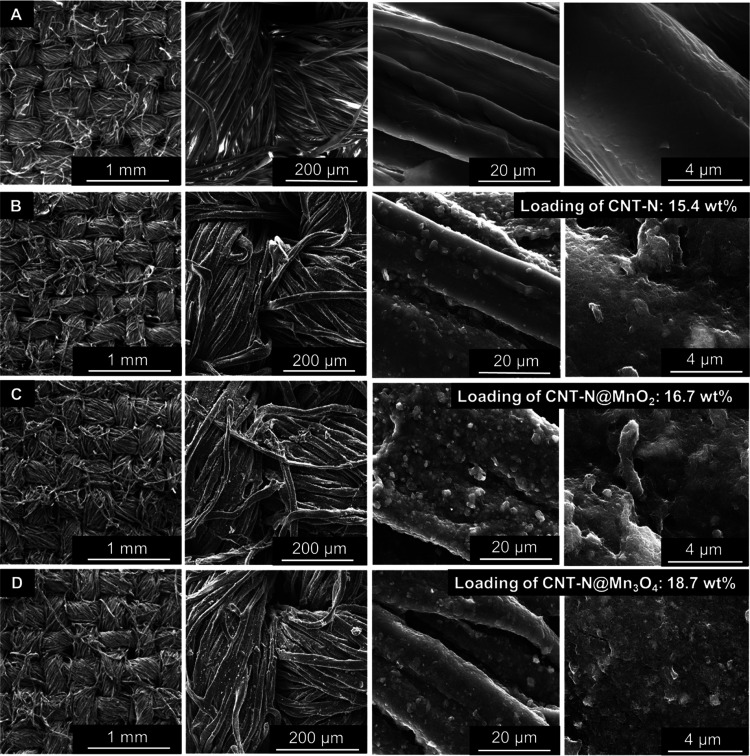
SEM images of the (A) original textile fabric and the fabric coated with (B) CNT-N, (C) CNT-N@MnO_2__CA, and (D) CNT-N@Mn_3_O_4__CA (from left to right: 100×, 500×, 5000×, and 50 000× magnification).

The SEM micrographs of the original cotton fabric (Figure 6A) show the presence of fibers with a regular mesh structure and a thickness of approximately 15 ± 4 μm, enriched in carbon and oxygen (48.7 and 50.4 atom %, respectively, by EDS, Table S3), with a residual amount of silicon (0.9 atom %, by EDS).

The micrographs of all coated fabrics (Figures 6B–D) reveal the presence of a rough coating over the surface of the fibers, composed of CNT bundles with the characteristic tubular morphology, confirming the incorporation of the nanomaterials. The MOs are not directly observed due to the resolution limit of the SEM technique. The SEM images also reveal that the structure of the cotton fibers after the impregnation process was preserved.

The EDS characterization of the coated textiles (Table S3) reveals an increase of the carbon atom % and a decrease of the oxygen loading ongoing from the pristine cotton fabric to the coated textiles (atom % C: 48.7% vs. 71.1–75.7%; atom % O: 50.4% vs. 18.9–24.2%) arising from the incorporated carbon-based nanomaterials. The presence of MO in the cotton fabrics coated with the hybrids is confirmed through the detection of Mn (2.4 and 2.0 atom % for cotton_CNT-N@MnO_2__CA and cotton_CNT-N@Mn_3_O_4__CA, respectively), which is uniformly distributed throughout the sample surface, as observed by elemental mapping (Figure S6, exemplified for CNT-N@Mn_3_O_4__CA). A small amount of sodium was detected in all coated fabrics (0.6–1.1 atom %), being assigned to the surfactant used in the preparation of the CNT-N-based aqueous dispersions (sodium cholate). The trace amounts of Al and Ti that were detected may arise from the commercial CNT and the sonication probe, respectively.

### 3.4 Electrochemical Performance of Smart Textile SCs

Asymmetric sandwich-type textile SCs were produced using one electrode of CNT-N-coated textile and the other electrode of cotton coated with the hybrid nanomaterial (CNT-N@MnO_2__CA or CNT-N@Mn_3_O_4__CA); PVA/H_3_PO_4_ solid-gel electrolyte was sandwiched between the two electrodes. The devices were denoted as CNT-N//CNT-N@MnO_2__CA and CNT-N//CNT-N@Mn_3_O_4__CA. For comparison, symmetric devices were also prepared, being denoted as CNT-N//CNT-N, CNT-N@MnO_2__CA//CNT-N@MnO_2__CA, and CNT-N@Mn_3_O_4__CA//CNT-N@Mn_3_O_4__CA, respectively. For each asymmetric SC, two different configurations were tested: (i) the CNT-N-coated fabric acting as the positive electrode and the hybrid-based textile working as the negative electrode and (ii) the opposite configuration. The “+” signal will be assigned to the positive electrode and the “–” signal to the negative one. The electrochemical performance of all SCs was evaluated by EIS, CV, and GCD techniques, in a standard two-electrode configuration.

The overall electrochemical performance of the asymmetric textile SCs was superior to that of the corresponding symmetric devices, and thus will be the focus of this work. The results obtained in the electrochemical evaluation of the symmetric devices are presented in Table S4 and Figure S7.

#### 3.4.1 EIS

**Figure 7:**
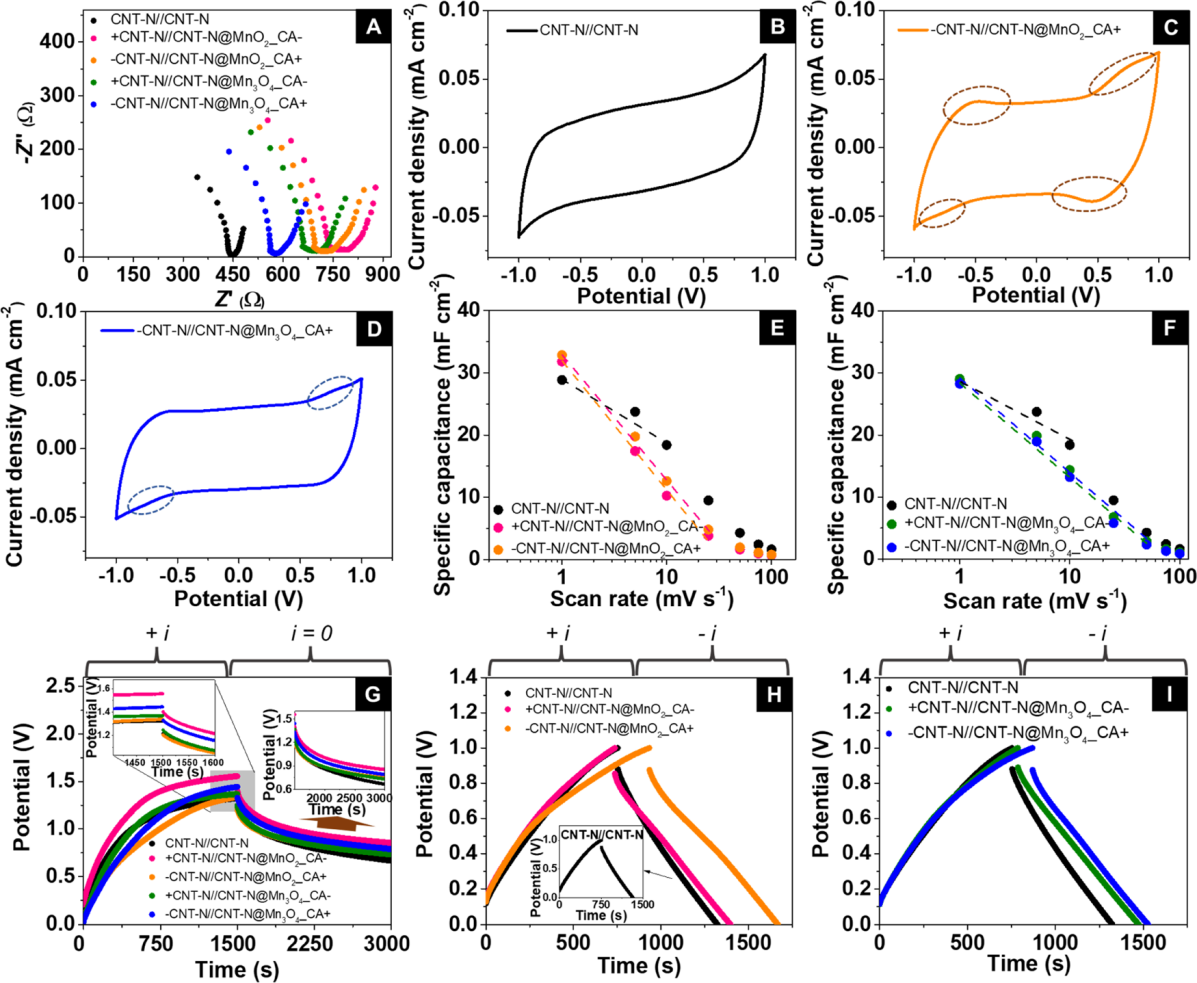
Electrochemical performance of symmetric CNT-N//CNT-N and asymmetric devices: (A) Nyquist plots; (B–D) *i*–*V* curves, at 1 mV s^–1^, in the range of −1.0 to 1.0 V; (E, F) areal capacitance as a function of the potential scan rate; (G) GCD curves with charging performed at 0.1 mA cm^–2^ and self-discharge under open circuit; and (H, I) charge/discharge curves at 0.10 mA cm^–2^. Inset of (G): Magnification of the *IR* drop region (left side) and discharge curves (right). Inset of (H): Charge/discharge curve of CNT//CNT at 0.1 mA cm^–2^.

The Nyquist plots of CNT-N//CNT-N, CNT-N//CNT-N@MnO_2__CA, and CNT-N//CNT-N@Mn_3_O_4__CA SCs (Figure 7A) were collected in the frequency range of 0.1 Hz to 1.0 MHz. The EIS curves of all SCs present similar profiles, being divided into three regions: a semicircle in the high-frequency zone (left side of the Nyquist plot, low real impedance values, *Z*’), which is related to the resistive contribution from the electrodes, the electrolyte, the contact resistance between the electrodes and the current collectors, and the electrode/electrolyte interfaces;^59^ a straight line with a slope of ∼45° at intermediate frequencies (Warburg impedance region) resulting from the combination of both resistive and capacitive phenomena associated with the penetration and diffusion of the electrolyte ions within the electrodes;^60^ finally, a second straight line at low frequencies (right side of the Nyquist plot), with a slope of 90°, which is related with the capacitive behavior of the devices, being attributed to the formation of an electric double-layer on both electrode/electrolyte interfaces.^59^

The Nyquist plots of the asymmetric devices present a larger semicircle at higher frequencies (larger radius) than that of the plot of the symmetric CNT-N//CNT-N, suggesting a higher resistance to the electrolyte ions diffusion, which can be related to the resistive behavior of the MOs anchored onto CNT-N. The straight line at low frequencies with a slope of ∼90° observed for all SCs corroborates the occurrence of the EDL-type mechanism within the devices assigned to the CNT material.

The equivalent series resistance (*R*_ES_) values of the SCs were extracted from the corresponding *Z*’ values at a frequency of 1 kHz.^59^ The *R*_ES_ values of the asymmetric SCs increase in the following order (Table 3): CNT//CNT (427 ± 11 Ω) < −CNT-N//CNT-N@Mn_3_O_4__CA+ (528 ± 4 Ω) < +CNT-N//CNT-N@Mn_3_O_4__CA– (603 ± 24 Ω) < −CNT-N//CNT-N@MnO_2__CA+ (637 ± 3 Ω) < +CNT-N//CNT-N@MnO_2__CA– (664 ± 11 Ω), following the same order of the areal electrical resistance of the corresponding textile electrodes (18.5 ± 0.3 Ω cm^–2^ for cotton_CNT-N, 35.8 ± 0.9 Ω cm^–2^ for cotton_CNT-N@Mn_3_O_4__CA, and 50.2 ± 2.9 Ω cm^–2^ for cotton_CNT-N@MnO_2__CA).

**Table 3:** Electrochemical Properties of CNT-N-Based Textile SCs

device	*R*_ES_ (Ω)	*R*_ESfit_^a^ (Ω)	*C*_A_^b^ (mF cm^–2^)	*V*_0_^c^ (V)	*IR* drop (V)	energy density^d^ (μW h cm^–2^)	power density^d^ (μW cm^–2^)	*V*_1500 s_^e^ (V)	energy density^f^ (μW h cm^–2^)	power density^f^ (μW cm^–2^)
CNT-N//CNT-N	427 ± 11	398	28.85	1.22	0.11	5.93	346.58	0.67	1.80	105.05
+CNT-N//CNT-N@MnO_2__CA–	664 ± 11	746	31.81	1.40	0.15	8.70	309.01	0.85	3.19	113.43
–CNT-N//CNT-N@MnO_2__CA+	637 ± 3	782	32.85	1.21	0.13	6.64	219.04	0.75	2.57	84.71
+CNT-N//CNT-N@Mn_3_O_4__CA–	603 ± 24	683	29.06	1.25	0.12	6.28	257.88	0.73	2.15	88.37
–CNT-N//CNT-N@Mn_3_O_4__CA+	528 ± 4	547	28.26	1.33	0.11	6.94	334.82	0.79	2.45	118.20

a Equivalent series resistance value obtained from the fitting of the EIS data.

b Areal specific capacitance of the SC, at a scan rate of 1 mV s^–1^, obtained from eq 1.

c Operation potential obtained by the GCD technique after a charging period of 1500 s with an applied current density of 0.1 mA cm^–2^.

d Energy density and power density values obtained from eqs 2 and 3, respectively, considering a scan rate of 1 mV s^–1^ and the *V*_0_ value after 1500 s of charging.

e Working potential obtained by the GCD technique after a discharging period of 1500 s under open circuit.

f Energy density and power density values obtained from eqs 2 and 3, respectively, considering a scan rate of 1 mV s^–1^ and the *V*_1500 s_ value after 1500 s of discharge under open circuit.

These results also highlight the importance of assembling asymmetric SCs, when compared to the symmetric counterparts, since the former present 2.1–2.7× lower *R*_ES_ values than the latter (637–664 Ω for CNT-N//CNT-N@MnO_2__CA vs. 1724 ± 10 Ω for CNT-N@MnO_2__CA//CNT-N@MnO_2__CA; 528–603 Ω for CNT-N//CNT-N@Mn_3_O_4__CA vs. 1090 ± 4 Ω for CNT-N@Mn_3_O_4__CA//CNT-N@Mn_3_O_4__CA, Figure S7 and Table S4). This improvement is due to the higher electrical conductivity of the CNT-N-based electrode relative to that of the hybrid MO-based electrode. Additionally, the *R*_ES_ values are higher for the MnO_2__CA-based devices than for the Mn_3_O_4_-based counterparts, which can be justified by the higher MO loading in the CNT-N@MnO_2__CA hybrid (surface atom % Mn: 3.5% vs. 1.2% for CNT-N@Mn_3_O_4__CA).

Concerning the influence of the polarity of the electrodes in the performance of the asymmetric devices, the preferential polarity that leads to lower *R*_ES_ values is when the CNT-N-based electrode is the negative (−) electrode and the hybrid-based electrode works as the positive (+) one.

**Figure 8:**
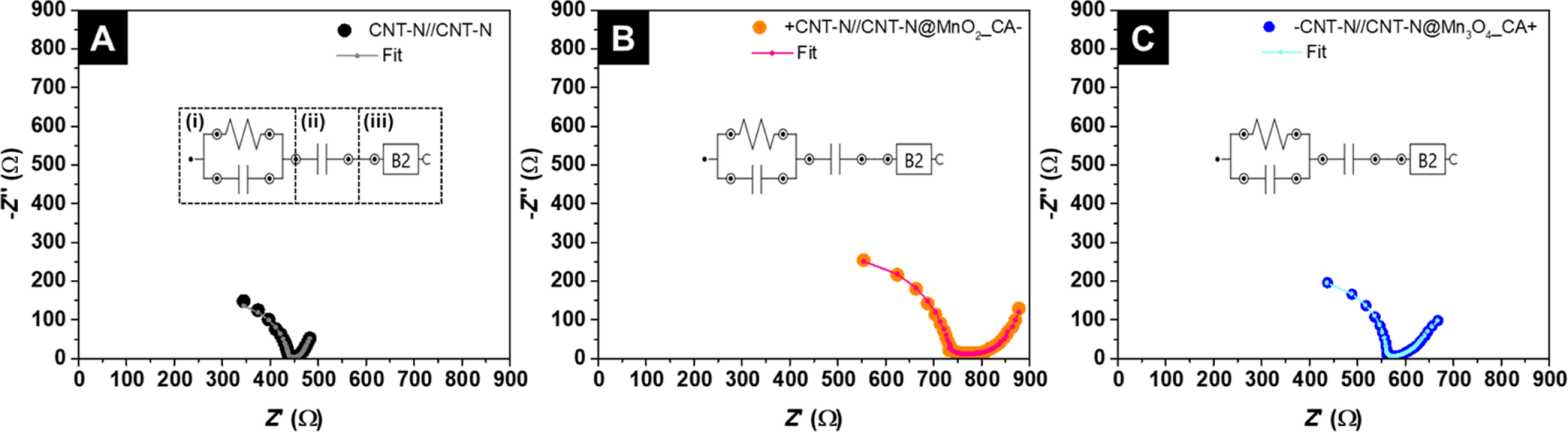
Nyquist plots with experimental data (black, orange, and blue points) and the respective fits (gray, pink, and ocean blue dash–dotted lines) of (A) CNT-N//CNT-N, (B) +CNT-N//CNT-N@MnO_2__CA–, and (C) −CNT-N//CNT-N@Mn_3_O_4__CA+ devices and the respective equivalent circuits (inset).

The raw frequency-dependent impedance data of each device was fitted considering three modules (see insets of the Nyquist plots presented in Figure 8): module (i) is composed of a resistance and a capacitor connected in parallel and is associated with the ionic resistance of the SC; module (ii) is a capacitor connected in series, which corresponds to the capacitance of the whole SC device; module (iii) is a B2 (Bisquert #2) component, based on the transmission line model, which takes into account the “porosity” of the fabric and/or CNT-N/MO electrode materials.^61^

To perform the fitting of the EIS data, the experimental values of *R*_ES_ and capacity (in *F*, determined by CV at 1 mV s^–1^, discussed in Section 3.4.2) were used as starting condition parameters to guide the fit parameters to reach the optimized solution. The theoretical *R*_ES_ values of all devices were extracted from the corresponding fitted Nyquist plots (denoted as *R*_ESfit_) and are summarized in Table 3. The *R*_ESfit_ values are comparable to the experimental *R*_ES_ results at *f* = 1 kHz, following the same variation tendency (CNT-N//CNT-N: 398 Ω vs. 427 Ω; +CNT-N//CNT-N@MnO_2__CA–: 746 Ω vs. 664 Ω; −CNT-N//CNT-N@MnO_2__CA+: 782 Ω vs. 637 Ω; +CNT-N//CNT-N@Mn_3_O_4__CA–: 683 Ω vs. 603 Ω; −CNT-N//CNT-N@Mn_3_O_4__CA+: 547 Ω vs. 528 Ω).

The capacitance of module (i) presents a residual value (order of pF); the existence of such capacitance is probably related to the textural properties of the textile fabric substrate and/or the substrate/electrode material interfaces.

#### 3.4.2 CV

**Scheme 1:**
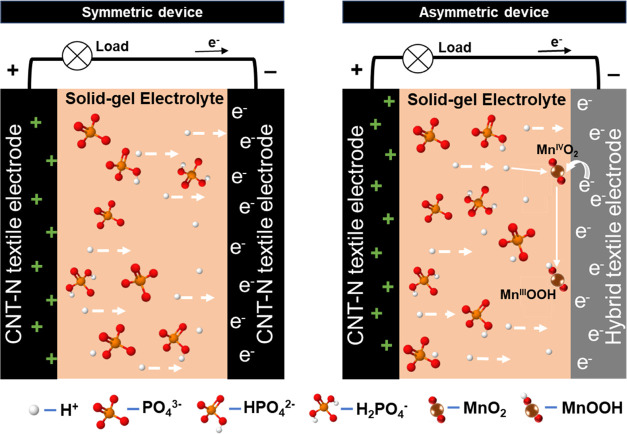
Energy Storage Working Mechanisms in the CNT-N//CNT-N Symmetric SC (left) and CNT-N//CNT-N@MnO_2__CA Asymmetric SC (right)

First, CV studies at 1 mV s^–1^ and different potential windows were performed for all textile SCs in order to identify the maximum potential window of each device (Figure S8). The *i*–*V* curves of all textile SCs, acquired at a scan rate of 1 mV s^–1^ in the respective maximum potential window, are presented in Figure 7B–D. The *i*–*V* curve of the symmetric CNT-N//CNT-N device (Figure 7B) exhibits a quasi-rectangular shape, which is characteristic of EDLCs with a nonfaradaic energy storage mechanism based on the reversible adsorption/desorption of electrolyte ions at the surface and/or within the pores of the electrode material (Scheme 1).^62^ No redox peaks are detected, despite the presence of nitrogen-based groups with potential redox-active properties (*i.e*., pyridinic and pyrrolic-based groups) in the CNT structure.^10^

In the case of the asymmetric SCs, the quasi-rectangular shape of the *i*–*V* cycles is preserved (Figure 7C,D). The *i*–*V* cycle of −CNT-N//CNT-N@MnO_2__CA+ (Figure 7C) evidences two humps centered at −0.49 ± 0.01 and 0.72 ± 0.01 V in the anodic sweep (positive current density range, highlighted with dashed circles in Figure 7C) and two humps at −0.76 ± 0.01 and 0.47 ± 0.01 V in the cathodic sweep (negative current density range). The presence of these humps is a signature of the occurrence of reversible oxidation–reduction reactions on/near the surface of the CNT-N@MnO_2__CA electrode material involving Mn^4+^ and Mn^3+^ cations, accompanied by the adsorption/desorption of the electrolyte ions (Scheme 1).^63^ According to the literature, this process can be expressed by the following equation^63^
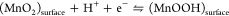
6

The *i*–*V* cycle of the −CNT-N//CNT-N@Mn_3_O_4__CA+ device (Figure 7D) displays two smooth humps centered at −0.76 ± 0.01 and 0.78 ± 0.01 V in the anodic and cathodic scans (highlighted with dashed circles in Figure 7D), corresponding to the oxidation of Mn^2+^ to Mn^3+^ and reduction of Mn^3+^ to Mn^2+^ within the grafted Mn_3_O_4_.^64^ In the literature and to the best of our knowledge, the energy storage mechanism of Mn_3_O_4_ electrode material has been mostly described in alkaline electrolytes. Nevertheless, Singh et al. suggested the occurrence of the dismutation of Mn_3_O_4_ in acidic electrolytes,^65^ which can be tentatively described by the following equation
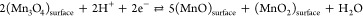
7

The *i*–*V* cycles of the asymmetric devices with opposite polarities are presented in Figure S9. Thus, the presence of both nonfaradaic electric double-layer-type and pseudocapacitive charge storage mechanisms, endowed by the CNT-N support and redox-active MOs within the hybrids, respectively, allows classifying the asymmetric devices as hybrid SCs.

The specific capacitance (*C*_A_) values of all devices were determined at 1 mV s^–1^ through eq 1 and are summarized in Table 3. The *C*_A_ values of the CNT-N//CNT-N@MnO_2__CA and CNT-N//CNT-N@Mn_3_O_4__CA asymmetric devices are 2.0–13.9% higher than those of CNT-N//CNT-N (CNT-N//CNT-N@MnO_2__CA: 31.81 and 32.85 mF cm^–2^; CNT-N//CNT-N@Mn_3_O_4__CA: 29.06 and 28.26 mF cm^–2^; CNT-N//CNT-N: 28.85 mF cm^–2^), which arises from the simultaneous occurrence of EDL-type and pseudocapacitive energy storage mechanisms. When comparing both asymmetric devices, the *C*_A_ values of the SC based on CNT-N@MnO_2__CA are up to 16.2% higher than those of the device based on CNT-N@Mn_3_O_4__CA, which can be related to the higher MnO_2__CA loading within CNT-N@MnO_2__CA, and/or to the distinct nanosheet/nanoflake-like morphology (vs. quasi-spherical shape of the grafted Mn_3_O_4__CA NPs), and/or to the higher oxidation states of Mn cations within MnO_2_ (+3 and +4 vs. +2 and +3 for Mn_3_O_4_). For each asymmetric SC, the similarity between the *C*_A_ values obtained for both configurations demonstrates their independence on the type of configuration used (*i.e*., electrode polarity).

The CV measurements were performed at different scan rates ranging from 1 to 100 mV s^–1^ and the respective *C*_A_ values were calculated (Figure 7E,F). There is a linear decrease of the *C*_A_ values until a scan rate of up to 10 mV s^–1^ for CNT-N//CNT-N, up to 25 mV s^–1^ for CNT-N//CNT-N@MnO_2__CA, and up to 50 mV s^–1^ for CNT-N//CNT-N@Mn_3_O_4__CA, indicating that the devices are more stable at lower scan rates, since the electrolyte ions have more time to penetrate within the electrode material at such scan rates, forming the electric double-layer required for the SCs to store energy.^39^

For each device, the capacitive and diffusion coefficients were calculated using Dunn’s equation^66^
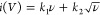
8where *i* is the current intensity at a specific potential, ν is the potential scan rate, *k*_1_ and *k*_2_ are proportionality constants at a fixed potential, *k*_1_*v* is the (pseudo)capacitive nondiffusion-limited contribution, and *k*_2_√*v* is the faradaic diffusion-limited contribution. The (pseudo)capacitive nondiffusion-limited contribution is assigned to the occurrence of adsorption/desorption of charges and fast redox reactions at the electrode/electrolyte interfaces and electrode materials surface. On the other hand, the faradaic diffusion-limited term is related to the kinetic limitations associated with the diffusion of the electrolyte ions and faradaic redox reactions within the electrode material (inner surface).^67^

**Figure 9:**
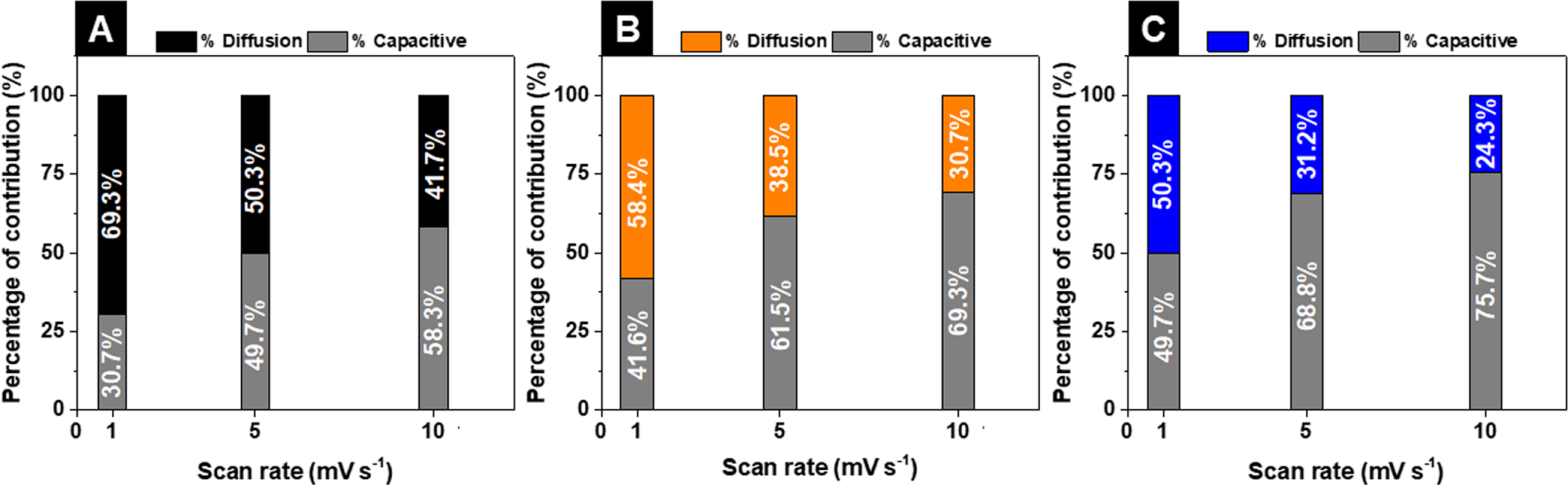
Bar charts with the contribution from capacitive- and diffusion-controlled processes at different scan rates for (A) CNT-N//CNT-N, (B) +CNT-N//CNT-N@MnO_2__CA–, and (C) −CNT-N//CNT-N@Mn_3_O_4__CA+ devices.

The contributions from the capacitive- and diffusion-controlled processes to the energy storage mechanism of each SC were extracted from the corresponding *i*(*V*)·(√*v*^–1^) vs. √*v* plot. The bar charts with both capacitive- and diffusion-controlled contributions (expressed in %) for all devices at specific potential scan rates are presented in Figure 9.

For all SCs, the capacitive-controlled contribution increases upon the increase of the potential scan rate, in accordance with the literature.^67^ Moreover, at each scan rate, the capacitive contribution is higher for the asymmetric SCs than for the symmetric device. For instance, at 1 mV s^–1^, the capacitive contribution increases from 30.7% for the symmetric CNT-N//CNT-N device to 41.6 and 49.7% for +CNT-N//CNT-N@MnO_2__CA– and −CNT-N//CNT-N@Mn_3_O_4__CA+, respectively. This enhancement can be justified by the additional contribution from the manganese oxide component to the energy storage process. Moreover, when comparing both asymmetric SCs, the presence of MnO_2__CA nanoparticles within the CNT-N@MnO_2__CA electrode material leads to an enhancement of the contribution from the diffusion-limited process of the resulting device, probably due to the more open wrinkled morphology and higher loading of electrochemically active MnO_2__CA species (vs. the quasi-spherical Mn_3_O_4__CA grafted onto CNT-N@Mn_3_O_4__CA).

#### 3.4.3 GCD

GCD measurements were performed by charging the devices at a current density of 0.1 mA cm^–2^ for 1500 s, in order to ensure that their potential reached stabilization at the end of the charging step, followed by the switching off of the applied current density to monitor their discharging behavior (Figure 7G). All devices presented similar profiles during charging: a faster charging in the first ∼8 min, followed by a slower charging process until reaching potential stabilization. The faster initial charging is due to the fact that, in the beginning of the process, the electrolyte ions have all of the surface area of the electrodes available to occupy, while for higher charging times, the available surface area reduces, resulting in slower charging.

After switching off the applied current density, a jump in the potential value is observed, being denoted as *IR* drop. Considering that the *IR* drop corresponds to the difference between the potential values immediately before and just after switching off the load charge, this value is related to the internal resistance of the devices. The *IR* drop values vary between 0.11 and 0.15 V (Table 3), demonstrating the low internal resistance of the textile SCs.

The operating potential (*V*_0_) values of all SCs, which correspond to the first potential value measured after switching off the applied current density of the SCs, are summarized in Table 3.^39^ In general, the use of CNT-N@MO hybrids as the active electrode material leads to SCs with enhanced *V*_0_ values (except for −CNT-N//CNT-N@MnO_2__CA+, with comparable *V*_0_ to that of CNT-N//CNT-N). In particular, +CNT-N//CNT-N@MnO_2__CA– and CNT-N@Mn_3_O_4__CA in both configurations present 14.8% and up to 9.0% higher *V*_0_ values than that of CNT-N//CNT-N (1.40 and 1.25–1.33 V, respectively vs. 1.22 V).

Both asymmetric devices present slower discharge under open circuit than CNT-N//CNT-N (right inset of Figure 7G), with the slowest self-discharge being observed for +CNT-N//CNT-N@MnO_2__CA–, demonstrating the importance of the hybrid electrode nanomaterials as well as the asymmetric configuration to develop SCs with enhanced performance. After 1500 s of discharge under open circuit, the difference between the working potential values of the asymmetric vs. symmetric devices becomes even larger (Table 3), with *V*_1500s_ of the asymmetric SCs being up to 26.9% higher than that of CNT-N//CNT-N.

Charge/discharge tests were also performed by applying different positive current density values (0.10, 0.15, and 0.20 mA cm^–2^) until the potential reached 1.0 V, followed by the corresponding negative current density values (Figures 7H,I and S10). The charge/discharge curves of CNT-N//CNT-N at different current densities present a triangular shape, which is characteristic of EDL-type mechanism endowed by the carbon nanomaterial. On the other hand, in the case of the asymmetric devices, the charge/discharge curves exhibit nonlinear profiles due to their hybrid energy storage mechanism (nonfaradaic and pseudocapacitive), in accordance with CV results.^59^ More specifically, when comparing the charge/discharge curves at 0.10 mA cm^–2^ of all devices (Figure 7H,I), the CNT-N//CNT-N SC requires 753 s to reach the cutoff value, being the device that charges and discharges faster (569 s). Among the asymmetric devices, +CNT-N//CNT-N@MnO_2__CA– presents a charging time (736 s) similar to that of CNT-N//CNT-N and the second lowest discharge time (659 s). −CNT-N//CNT-N@MnO_2__CA+ and the asymmetric CNT-N@Mn_3_O_4__CA-based devices (for both polarities) require up to 1.24× more time to reach the maximum potential value (934 and 786–869 s, respectively) and up to 1.29× more time to discharge (733 and 655–686 s, respectively) than CNT-N//CNT-N. Interestingly, the asymmetric devices with the CNT-N-coated textile as the negative electrode require more time to charge (slower charging profiles) than the opposite configuration. In all cases, the charge/discharge profiles are faster at higher current density values (Figure S10).

In order to assess the stability of all textile SCs upon multiple charge/discharge cycles, 8000 *i*–*V* cycles were performed by CV at 10 mV s^–1^ (Figure S11). All SCs retain 96–99% of the initial capacitance after 8000 charge/discharge cycles, proving their high cycling stability. After the cycling tests, all devices were characterized by XRD, Raman spectroscopy, and SEM. The X-ray diffractograms, Raman spectra, and SEM images of the electrode section of all SCs after the cycling tests are similar to those of the corresponding textile electrodes before the device assembly (Figures S12 and S13), confirming the robustness and stability of the devices.

**Figure 10:**
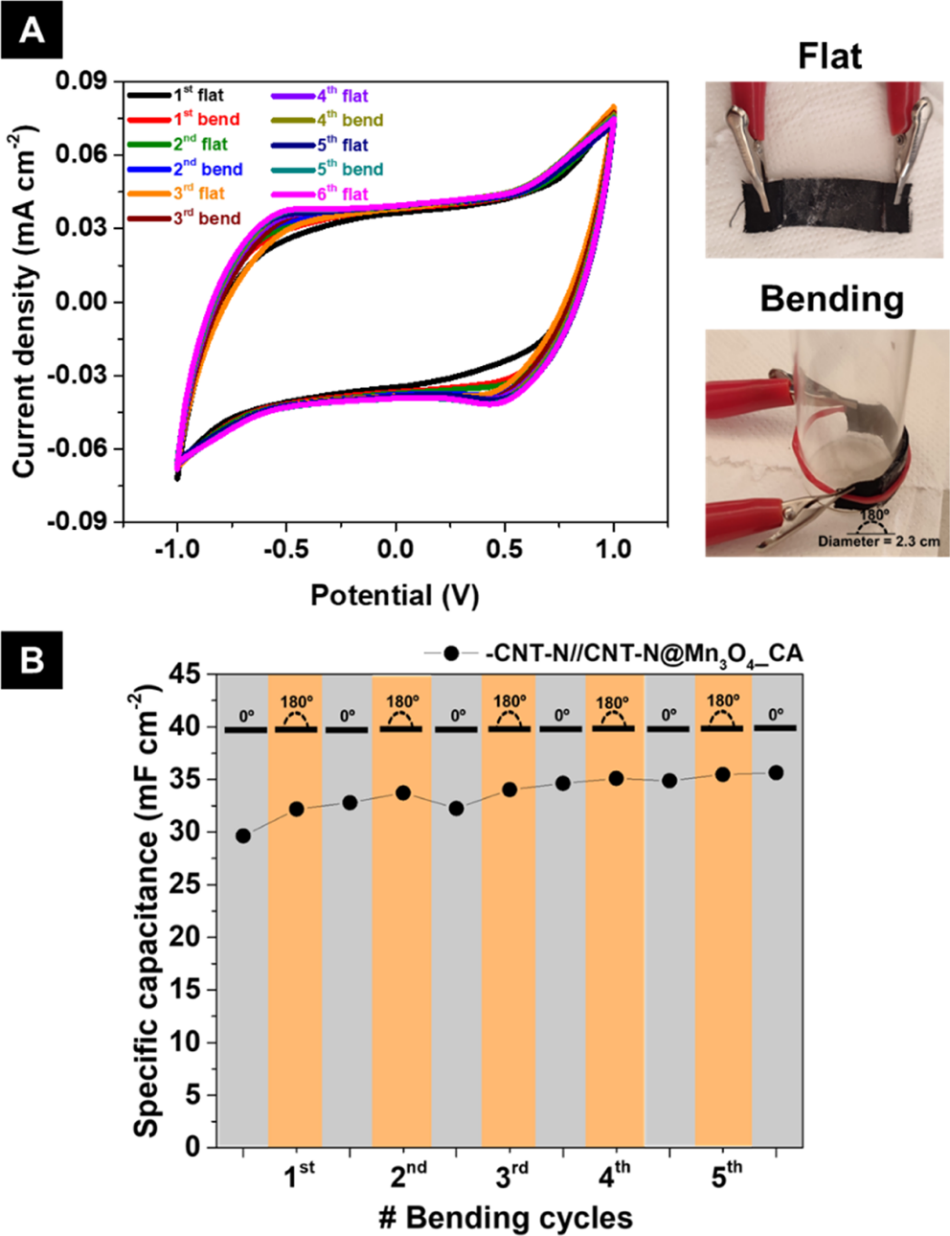
Flexibility studies for the CNT-N//CNT-N@Mn_3_O_4__CA device: (A) Cyclic voltammograms at 1 mV s^–1^ upon multiple bending/flat cycles and digital images of the device in the flat and bent positions; and (B) specific capacitance values upon multiple bending/flat cycles.

Bending tests were also performed for the CNT-N//CNT-N@Mn_3_O_4__CA device to evaluate its flexibility. The electrochemical performance of the device upon multiple bending cycles was monitored by the CV technique at a scan rate of 1 mV s^–1^ (Figure 10A). The textile-based SC maintained its performance after at least five bending cycles, with a specific capacitance variation below 3% (Figure 10B), confirming its flexibility.

In order to unveil the influence of the type of connection between SC units, two CNT-N@Mn_3_O_4__CA SCs (2.5 × 1.0 cm^2^ each) were connected in series or in parallel, and GCD and CV tests were performed. The GCD results (Figure S14A) demonstrate that the output potential of the devices connected in series is almost twice the value obtained for a single device under similar current density conditions (3.33 V vs. 1.33 V). Furthermore, the CV results (Figure S14B) show that the output current intensity of two devices assembled in parallel is 1.5× higher than the value of a single unit (0.20 mA vs. 0.13 mA).

#### 3.4.4 Energy Density and Power Density

**Figure 11:**
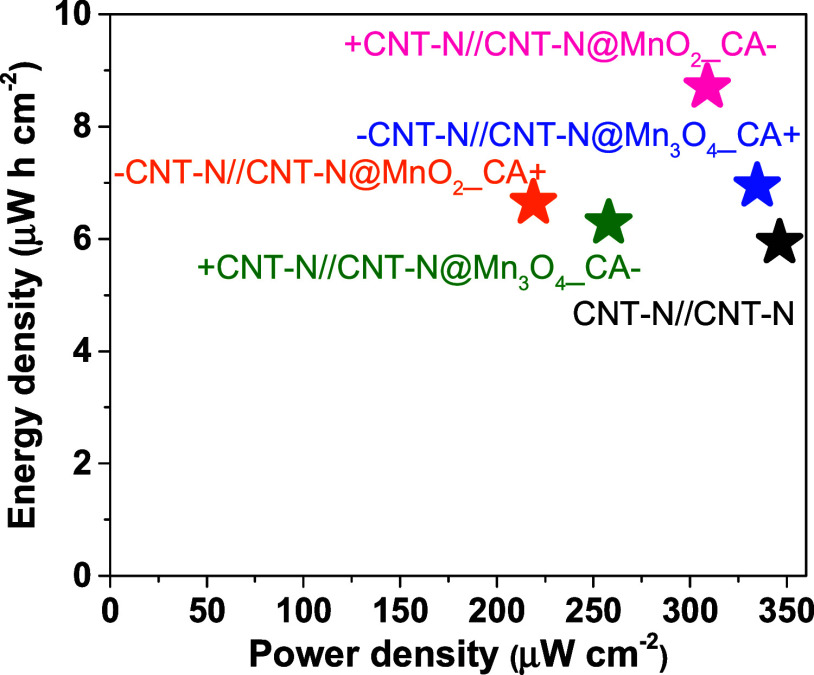
Ragone plot of areal energy density vs. areal power density for the CNT-N//CNT-N and asymmetric textile SCs prepared in this work.

For practical applications, the most useful parameters to evaluate the performance of energy storage systems are the energy density and power density. In this context, the energy density (*E*) and power density (*P*) of the assembled textile SCs were determined using eqs 2 and 3, respectively. The results are summarized in Table 3 and in the Ragone plot presented in Figure 11.

All asymmetric hybrid SCs present 5.9–46.7% enhancements in the energy density values relative to the CNT-N//CNT-N device. The +CNT-N//CNT-N@MnO_2__CA– device presents the best performance among all devices, with an energy density of 8.70 μW h cm^–2^, which is 46.7% higher than that of CNT-N//CNT-N (5.93 μW h cm^–2^) and with comparable power density of 309.01 μW cm^–2^ (vs. 346.58 μW cm^–2^ for CNT-N//CNT-N). These achievements can be related to (i) the higher MO loading within the CNT-N@MnO_2_ hybrid relative to CNT-N@Mn_3_O_4_, (ii) the higher oxidation states of manganese cations in MnO_2_ relative to Mn_3_O_4_ (+3 and +4 vs. +3 and +2), and/or (iii) the higher specific surface area of CNT-N@MnO_2_ and the wrinkled-type morphology of the grafted MnO_2_, which contribute to the adsorption of a higher amount of electrolyte ions.^68^

The energy density and power density values were also calculated considering the working potential of the devices after a self-discharge period of 1500 s (Table 3). In such a case, the energy density of the asymmetric devices in the most promising configurations becomes 36.1–77.2% higher than that of the symmetric device, and the power density values are 8.0–12.5% higher, further attesting their superior energy storage ability.

When comparing with textile SCs based on carbon/MO composite/hybrids recently reported in the literature (Table S5), the asymmetric textile SCs prepared in this work exhibit an improvement of up to 1.38× in the energy density and up to 2.25× in the power density (calculated using *V*_0_) relative to the values achieved by a symmetric SC based on hydrogenated ZnO@amorphous ZnO@MnO_2_ core–shell nanocables grown on conductive carbon cloth substrate, using PVA/LiCl solid-gel as the electrolyte (energy density: 69.59–90.00 μW h cm^–3^vs. 40.00 μW h cm^–3^; power density: 3.20–3.36 mW cm^–3^ vs. 2.44 mW cm^–3^).^69^ Additionally, when compared with textile SCs developed on nonconductive substrates, the devices prepared in this work present up to 1.68× higher power density than that of a symmetric SC based on textile electrodes of graphene/MnO_2_ deposited on a pure cellulose nonwoven substrate and PVA/H_2_SO_4_ electrolyte (0.335 mW cm^–2^ vs. 0.2 mW cm^–2^).^70^

### 3.5 Practical Application of Hybrid Textile SCs

**Figure 12:**
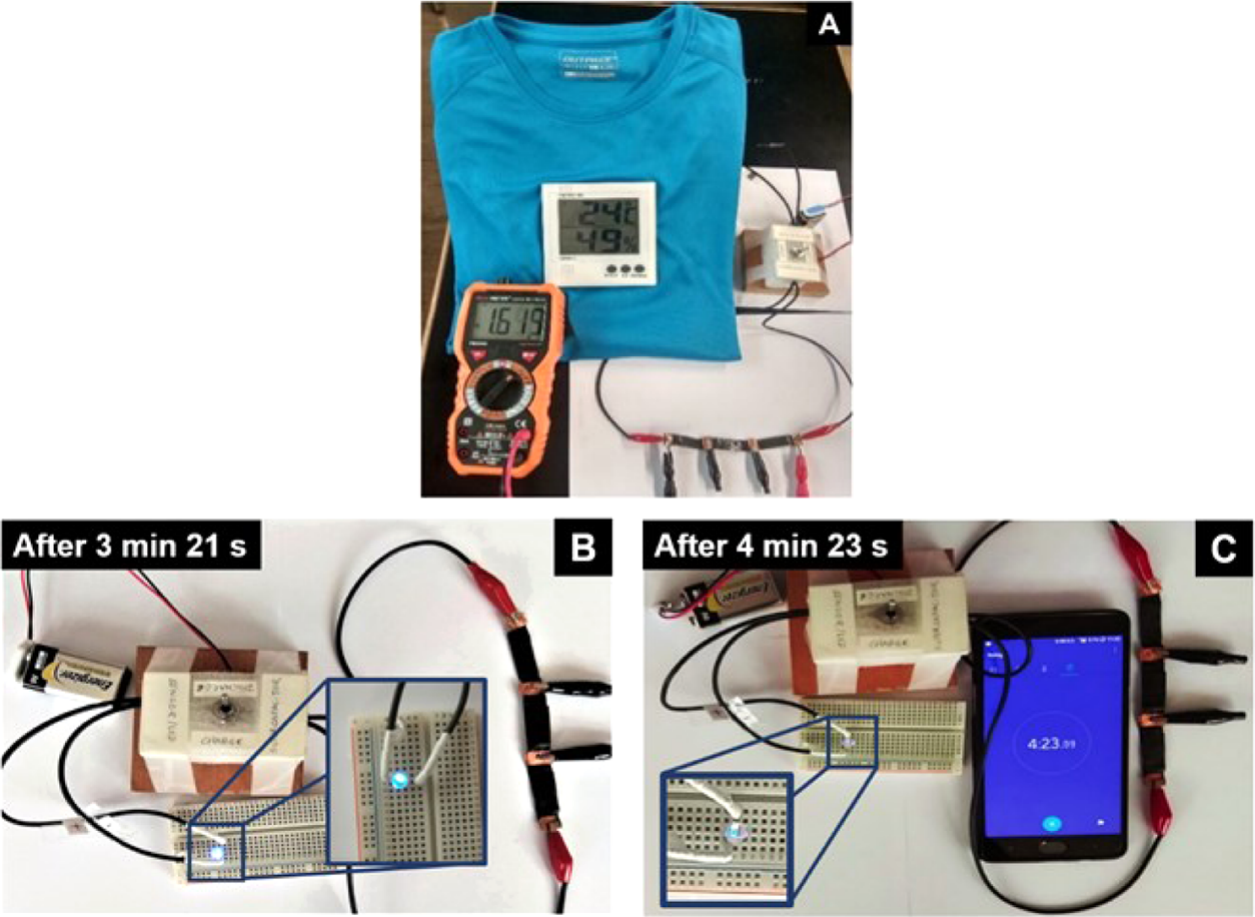
Proof-of-concept of the practical operating performance of three CNT-N//CNT-N@Mn_3_O_4__CA SCs (7.5 × 1.0 cm^2^) connected in series (A) to supply a humidity/thermometer digital sensor during 10 min and a blue LED (global operating potential of 2.76 V) during (B) 3 min 21 s and (C) 4 min 23 s of discharge.

To assess the real potential of these devices to power smart electronic devices, as a proof-of-concept, three CNT-N//CNT-N@Mn_3_O_4__CA SCs were connected in series (total active area of 7.5 cm^2^) and used to power a sensor and a blue light emitting diode (LED); see Figure 12. The set of three asymmetric SCs was able to maintain a humidity/thermometer digital sensor in operation for ∼10 min with a load of only 15 s (Figure 12A and Movie 1 in the Supporting Information). Additionally, the same SC assembly could feed a blue LED for 3 min 20 s, maintaining a residual light until ∼4 min 23 s, after only 10 s of charging (Figure 12B,C and Movie 2 in the Supporting Information).

## 4 Conclusions

CNT-N@MnO_2_ and CNT-N@Mn_3_O_4_ hybrid nanomaterials were prepared by a simple and cost-effective one-pot aqueous precipitation method at low temperatures, using KMnO_4_ as the MO precursor, MIPA as the mild alkaline agent, citric acid as the chelating agent, and adjustable temperature (room temperature or 100 °C), envisaging the tuning of the phase type, shape, and size of the anchored MO NPs. Remarkably, pure hausmannite was obtained at low temperatures (100 °C), without requiring a *post*-thermal treatment step. The MnO_2_ particles immobilized onto the CNT-N surface presented a hexagonal birnessite structure and wrinkle-like morphology, while the anchored Mn_3_O_4_ exhibited a spinel-type tetragonal hausmannite structure and quasi-spherical shape (particle size of ∼16 ± 4 nm, by TEM). For both hybrids, the MOs were preferentially anchored onto the CNT-N surface via its pyridinic-based functional groups.

Both sandwich-type asymmetric textile SCs, CNT-N//CNT-N@MnO_2__CA and CNT-N//CNT-N@Mn_3_O_4__CA, presented 5.9–46.7% enhancement in the energy density values relative to CNT-N//CNT-N, arising from the simultaneous occurrence of EDL-type and pseudocapacitive energy storage mechanisms. In particular, the +CNT-N//CNT-N@MnO_2__CA– device presented the best performance, with 46.7% higher energy density than that of CNT-N//CNT-N (8.70 μW h cm^–2^ vs. 5.93 μW h cm^–2^) and comparable power density (309.01 μW cm^–2^ vs. 346.58 μW cm^–2^). Moreover, it afforded up to 16% higher specific capacitance, up to 15% higher operation potential, and up to 47% higher energy density than those of the CNT-N//CNT-N@Mn_3_O_4__CA device, due to the higher MnO_2_ loading within the corresponding hybrid, the higher oxidation states of manganese cations within MnO_2_ and/or the distinct morphology of the MnO_2_ NPs. All SCs exhibited excellent cycling stability (>96% capacitance retention after 8000 charge/discharge cycles).

Hence, this work demonstrated that CNT-N@MnO_2_ and CNT-N@Mn_3_O_4_ hybrids are promising electrode nanomaterials for the design of solid-state energy storage textile devices. Moreover, it highlighted the versatility of the developed eco-friendly citric acid-promoted precipitation process to hybridize CNT-N nanomaterials with pure MO phases, opening promising prospects for a plethora of applications, including energy storage, (photo)catalysis, biosensing, environmental remediation, and electrochemical detection.

## Supporting Information Available

XPS characterization of CNT-N and manganese oxide-based nanomaterials; SEM characterization of manganese oxide nanomaterials; TEM characterization of CNT; EDS characterization of CNT-N-based nanomaterials, parent and coated textile fabrics; N_2_ adsorption–desorption isotherms of CNT-based materials; variation of electrical resistance and nanomaterial loading in the coated cotton fabrics vs. number of dip-pad-dry steps; electrochemical performance of symmetric CNT-N-based textile SCs; *i*–*V* cycles at 1.0 mV s^–1^ and different potential windows of the CNT-N-based textile SCs; *i*–*V* cycles of the asymmetric textile SCs with opposite polarities at 1.0 mV s^–1^; charge/discharge curves of the symmetric and asymmetric CNT-N-based devices at different current densities; cycling stability of CNT-based devices; characterization of the symmetric and asymmetric textile SCs after the cycling tests by XRD, Raman spectroscopy, and SEM; electrochemical performance of two asymmetric devices of CNT-N//CNT-N@Mn_3_O_4__CA connected in series or in parallel; and comparison of energy storage performance with the literature on Mn_*x*_O_*y*_-based textile SCs (PDF)
Videoclips showing the practical operational performance of the textile SCs in real operation conditions when powering an electronic sensor (Movie 1) (MP4)
Videoclips showing the practical operational performance of the textile SCs in real operation conditions when powering an LED (Movie 2) (MP4)
