# Supplementary material for: Fabrication of CNT-N@Manganese Oxide Hybrid Nanomaterials through a Versatile One-Pot Eco-Friendly Route toward Engineered Textile Supercapacitors
Source: ACS Appl Eng Mater. 2024 Mar 29;2(4):1170–89. doi: 10.1021/acsaenm.4c00164 (PMC11060322; doi:10.1021/acsaenm.4c00164)
Supplement: Supplementary file 1 — em4c00164_si_001.pdf [file em4c00164_si_001.pdf]

Supporting Information

Fabrication of CNT-N@Manganese Oxide Hybrid Nanomaterials through a Versatile One-Pot Eco-Friendly Route toward Engineered Textile Supercapacitors

Joana S. Teixeira[1,2], Rui S. Costa[1,2], Alexandra Guedes[3], André M. Pereira[2], Clara R. Pereira[1,*]

[1] REQUIMTE/LAQV, Departamento de Química e Bioquímica, Faculdade de Ciências, Universidade do Porto, Rua do Campo Alegre s/n, 4169-007 Porto, Portugal

[2] IFIMUP, Instituto de Física de Materiais Avançados, Nanotecnologia e Fotónica, Departamento de Física e Astronomia, Faculdade de Ciências, Universidade do Porto, Rua do Campo Alegre s/n, 4169-007 Porto, Portugal

[3] Instituto de Ciências da Terra – Pólo Porto, Departamento de Geociências, Ambiente e Ordenamento do Território, Faculdade de Ciências, Universidade do Porto, Rua do Campo Alegre s/n, 4169-007 Porto, Portugal

***Corresponding author:**

Dr. Clara R. Pereira: Tel: +351 220402576; Fax: +351 220402659; e-mail: clara.pereira@fc.up.pt

Table S1. Binding energies and areas of the components in the C 1s, O 1s, N 1s and Mn $2p_{3/2}$ core-level regions obtained by curve fitting of the XPS spectra of CNT-N and manganese oxide-based nanomaterials

Material	C 1s			O 1s			N 1s			Mn $2p_{3/2}$		
	BE[a] (eV)	Assign.	Area (%)[b]	BE (eV)[a]	Assign.	Area (%)[b]	BE (eV)[a]	Assign.	Area (%)[b]	BE (eV)[a]	Assign.	Area (%)[b]
**CNT-N**	284.6 (0.7)	C=C	60.4	532.0 (2.7)	O=C	61.4	398.6 (1.6)	Pyridinic N	54.8			
	285.1 (1.1)	C–C	11.2	533.7 (2.7)	O–C	38.6	400.2 (1.6)	Pyrrolic N	30.5			
	286.1 (1.1)	C–O/C=N	5.4				401.7 (1.6)	G/Q N[c]	10.1			
	287.1 (1.1)	C=O/C–N	3.4				404.2 (1.6)	Pyr-N-O[d]	4.6			
	288.1 (1.1)	O–C=O	1.8									
	290.6 (5.2)	π–π* sat.	17.8									
**CNT-N@MnO$_2$_CA**	284.6 (0.7)	C=C	62.9	530.0 (1.2)	Mn–O–Mn	73.4	398.7 (1.6)	Pyridinic N	51.9	641.2 (1.6)	Mn^{3+}	19.9
	285.2 (1.2)	C–C	8.6	531.2 (1.2)	Mn–OH	11.1	400.3 (1.6)	Pyrrolic N	38.1	642.4 (1.3)	Mn^{4+}	37.1
	286.1 (1.2)	C–O/C=N	5.2	532.2 (1.2)	O=C	9.3	402.0 (1.6)	G/Q N[c]	6.9	643.2 (1.3)	Mn^{4+}	19.5
	287.0 (1.2)	C=O/C–N	3.4	533.2 (1.2)	O–C	4.9	403.7 (1.6)	Pyr-N-O[d]	3.1	643.9 (1.3	Mn^{4+}	13.8
	288.2 (1.2)	O–C=O	2.0	534.4 (1.2)	Ads. H_2O	1.3				644.7 (1.3)	Mn^{4+}	5.2
	290.6 (5.9)	π–π* sat.	17.9							645.5 (1.3)	Mn^{4+}	3.1
										646.5 (1.3)	Mn^{4+}	1.4
**MnO$_2$_CA**	284.6 (1.4)	C–C/C–H	69.6	529.8 (1.2)	Mn–O–Mn	79.8	399.7 (1.8)	–NH_2[f]	100.0	641.0 (1.4)	Mn^{3+}	20.2
	286.2 (1.4)	C–OH/C–N[e,f]	16.6	531.2 (1.2)	Mn–OH	11.7				642.2 (1.1)	Mn^{4+}	36.9
	288.3 (1.4)	O–C=O[e]	13.8	532.2 (1.2)	O=C[e]	6.1				643.0 (1.1)	Mn^{4+}	19.5
				533.4 (1.2)	O–C[e,f]	2.5				643.7 (1.1)	Mn^{4+}	13.7
										644.5 (1.1)	Mn^{4+}	5.2
										645.3 (1.1)	Mn^{4+}	3.1
										646.3 (1.1)	Mn^{4+}	1.4

[a] The values between brackets refer to the full width at half-maximum (FWHM) of the bands.
[b] Area of each component in relation to the total area of the corresponding fitted core-level spectrum.
[c] G/Q N – graphitic/quaternary nitrogen.
[d] Pyr-N-O – pyridine-N-oxide.
[e] From citric acid.
[f] From MIPA alkaline agent.

Table S1. (cont.) Binding energies and areas of the components in the C 1s, O 1s, N 1s and Mn $2p_{3/2}$ core-level regions obtained by curve fitting of the XPS spectra of CNT-N and manganese oxide-based nanomaterials

Material	C 1s			O 1s			N 1s			Mn $2p_{3/2}$		
	BE[a] (eV)	Assign.	Area (%)[b]	BE (eV)[a]	Assign.	Area (%)[b]	BE (eV)[a]	Assign.	Area (%)[b]	BE (eV)[a]	Assign.	Area (%)[b]
CNT-N@Mn_3O_4_CA	284.6 (0.7)	C=C	62.0	530.5 (1.4)	Mn–O–Mn	37.0	398.6 (1.2)	Pyridinic N	45.9	640.9 (2.7)	Mn^{2+}	6.5
	285.1 (1.1)	C–C	10.4	531.6 (1.4)	Mn–OH	28.4	400.0 (1.2)	Pyrrolic N	34.3	641.3 (2.7)	Mn^{2+}	15.0
	286.3 (1.1)	C–O/C=N	6.1	532.7 (1.4)	O=C	25.2	401.6 (1.2)	G/Q N[c]	14.9	642.2 (2.7)	Mn^{2+}	5.9
	287.3 (1.1)	C=O/C–N	3.8	533.9 (1.4)	O–C	9.4	403.7 (1.2)	Pyr-N-O[d]	4.9	643.2 (2.7)	Mn^{2+}	3.4
	288.4 (1.1)	O–C=O	1.8							644.3 (2.7)	Mn^{2+}	1.3
	290.6 (1.1)	π–π* sat.	15.9							647.6 (2.7)	Mn^{2+} sat.	0.5
										640.8 (2.7)	Mn^{3+}	12.7
										641.9 (2.7)	Mn^{3+}	29.9
										643.2 (2.7)	Mn^{3+}	17.0
										644.7 (2.7)	Mn^{3+}	5.7
										646.3 (2.7)	Mn^{3+}	2.1
Mn_3O_4_CA	284.6 (1.4)	C–C/C–H	55.3	530.0 (1.5)	Mn–O–Mn	43.5	399.8 (1.1)	$–NH_2$[f]	79.8	640.7 (2.7)	Mn^{2+}	6.4
	286.2 (1.4)	C–OH/C–N[e,f]	14.8	531.4 (1.5)	Mn–OH	37.4	401.6 (1.1)	Protonated amine[f]	20.2	641.1 (2.7)	Mn^{2+}	14.9
	288.4 (1.4)	O–C=O[e]	29.9	532.4 (1.5)	O=C[e]	19.1				642.0(2.7)	Mn^{2+}	5.9
										643.0 (2.7)	Mn^{2+}	3.3
										644.1(2.7)	Mn^{2+}	1.3
										647.4 (2.7)	Mn^{2+} sat.	1.3
										641.0 (2.7)	Mn^{3+}	12.6
										642.1 (2.7)	Mn^{3+}	29.7
										643.4 (2.7)	Mn^{3+}	16.9
										644.9 (2.7)	Mn^{3+}	5.7
										646.5 (2.7)	Mn^{3+}	2.1

[a] The values between brackets refer to the full width at half-maximum (FWHM) of the bands.
[b] Area of each component in relation to the total area of the corresponding fitted core-level spectrum.
[c] G/Q N – graphitic/quaternary nitrogen.
[d] Pyr-N-O – pyridine-N-oxide.
[e] From citric acid.
[f] From MIPA alkaline agent.

Table S2. Binding energies and areas of the components in the Al 2p, K 2p and Si 2p core-level regions obtained by curve fitting of the XPS spectra of CNT-N and manganese oxide-based nanomaterials

Material	Al 2p		K 2p		Si 2p	
	BE (eV)[a]	Area (%)[b]	BE (eV)[a]	Area (%)[b]	BE (eV)[a]	Area (%)[b]
**CNT-N**	76.4 (3.2)	66.7				
	76.8 (3.2)	33.3				
**MnO_2_CA**			292.5 (1.2)	66.7	101.4 (1.1)	66.7
			295.3 (1.2)	33.3	102.0 (1.1)	33.3
**CNT-N@MnO_2_CA**			292.6 (1.1)	66.7		
			295.4 (1.1)	33.3		
**Mn_3O_4_CA**			293.0 (1.8)	66.7	102.1 (1.7)	66.7
			295.8 (1.8)	33.3	102.7 (1.7)	33.3
**CNT-N@Mn_3O_4_CA**	74.3 (1.6)	31.1			102.0 (1.5)	52.3
	74.7 (1.6)	15.5			102.6 (1.5)	26.1
	76.0 (1.6)	35.6			103.6 (1.5)	14.4
	76.4 (1.6)	17.8			104.2 (1.5)	7.2

[a] The values between brackets refer to the full width at half-maximum (FWHM) of the bands.
[b] Area of each component in relation to the total area of the corresponding fitted core-level spectrum.

Table S3. Atomic percentages of the different elements detected by EDS in the parent and coated textile fabrics

Fabric	Atomic%						
	**C**	**O**	**Mn**	**Al**	**Na**	**Si**	**Ti**
**Cotton**	48.7	50.4	–	–	–	0.9	–
**Cotton_CNT-N**	73.3	24.2	–	1.1	0.6	0.8	–
**Cotton_CNT-N@MnO_2_CA**	75.7	18.9	2.4	1.2	1.1	0.3	0.4
**Cotton_CNT-N@Mn_3O_4_CA**	71.1	23.3	2.0	0.9	1.0	0.6	0.9

Table S4. Electrochemical performance of the symmetric CNT-N-based textile SCs

**Devices**	R_{ES} (Ω)	**Specific capacitance**[a] **(mF cm^{-2})**	V_0 **(V)**	***IR* drop (V)**	**Energy density (μW h cm^{-2})**	**Power density (mW cm^{-2})**
**CNT-N//CNT-N**	427±11	28.85	1.22	0.11	5.93	0.347
**Sym_CNT-N@MnO_2_CA**	1720±10	24.45	0.98	0.31	3.27	0.056
**Sym_CNT-N@Mn_3O_4_CA**	1090±4	25.83	1.11	0.17	4.44	0.113

[a] Potential window of -1.0 → 1.0 V at 1 mV s^{-1}

Table S5. Comparison of the energy storage performance of the textile SCs prepared in this work with that of other Mn_xO_y-based textile SCs recently reported in the literature

Flexible substrate	Electrode material	Electrolyte	Energy density (μW h cm^{-2})	Energy density (μW h cm^{-3})	Power density (mW cm^{-2})	Power density (mW cm^{-3})	Ref.
**Cotton textile**	CNT//CNT	PVA/H_3PO_4	5.93	48.24	0.347	2.82	Our work
	+CNT-N//CNT-N@MnO_2_CA-		8.70	90.00	0.309	3.20	
	-CNT-N//CNT-N@Mn_3O_4_CA+		6.94	69.59	0.335	3.36	
**Pure nonwoven cellulose**	MnO_2/graphene	PVA/H_2SO_4	12.34	–	0.2	–	S1
**Cotton-derived CC[a]**	MnO_2@CC[a]//CC	Na_2SO_4	30.1	–	0.15	–	S2
			5.8	–	7.5	–	
**Carbon cloth**	Hydrogenated single-crystal ZnO@ZnO-doped MnO_2 core-shell nanocables	PVA/LiCl	–	40	–	2.44	S3
	Mn_3O_4//activated carbon	PVA/LiCl	25	–	0.64	–	S4
			14	–	3.25	–	
	Mn_3O_4/GA//CNH/GA[b]	PVA/NaOH	17.4	–	14.1	–	S5
**Carbon fiber cloth**	MnO_2/CNT	KOH	11.1	–	0.933	–	S6
			6.3	–	8.028	–	
**CNT fiber**	MnO_2/PtNP/CNT[c]	PVA/LiCl	1.84	–	0.0133	–	S7
**Free-standing film**	MnO_2@rGO	PPA/KOH[d]	1.01	–	0.12	–	S8
	Mn_3O_4/rGO/SWCNT	PVA/KOH	32	–	0.392	–	S9

[a] CC – Carbon cloth; [b] GA – 3D graphene aerogels; CNH – carbon nanohorns; [c] PtNP – Platinum nanoparticles; [d] PPA – potassiumpolyacrylate.

Figure S1. SEM images of (A) MnO_2_CA and (B) Mn_3O_4_CA with 100000× (top) and 200000× (bottom) magnification.

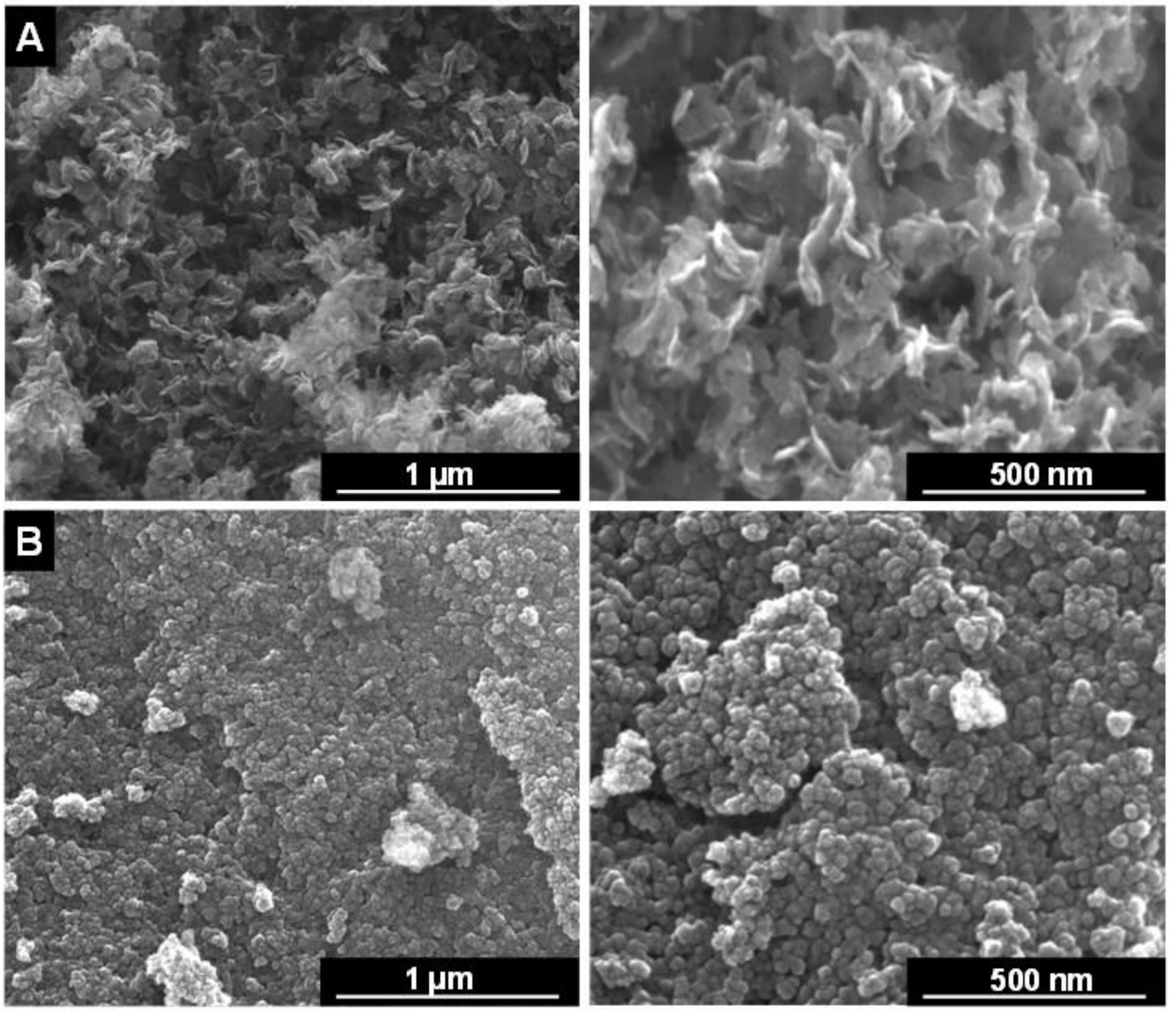

Figure S2. TEM image of the pristine CNT.

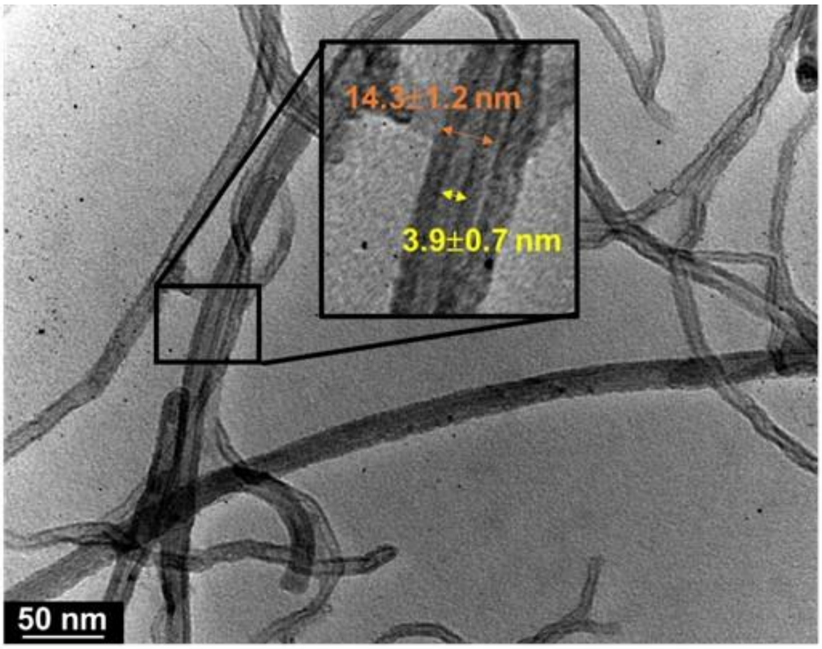

Figure S3. EDS spectra in TEM mode for all CNT-N-based nanomaterials.

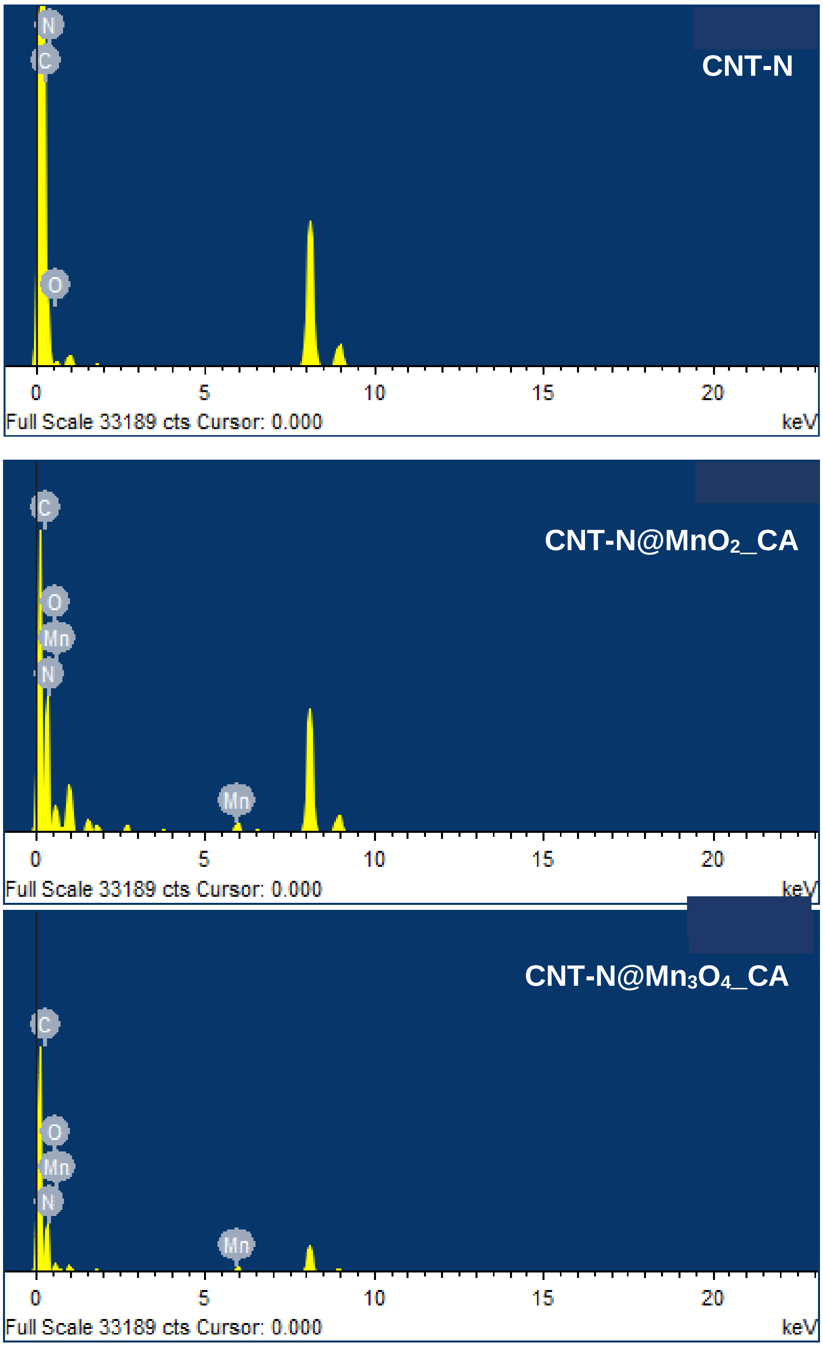

Figure S4. N_2 adsorption-desorption isotherms at -196 °C of the CNT-based samples: (A) CNT, (B) CNT-N, (C) CNT-N@MnO_2_CA and (D) CNT-N@Mn_3O_4_CA.

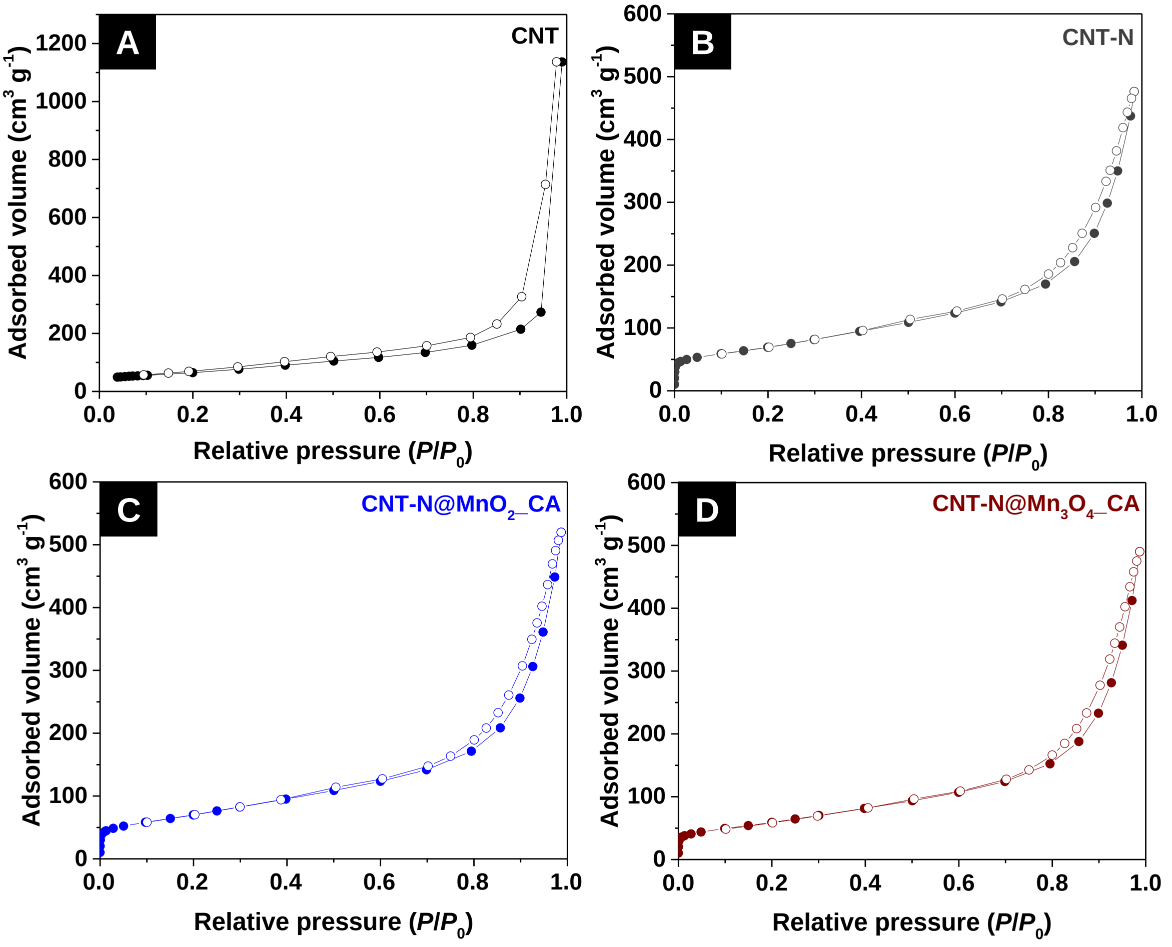

Figure S5. Variation of (A) specific electrical resistance (in Ω mg^{-1}) and (B) amount of incorporated CNT-N-based material in the coated cotton fabrics (in wt%) *vs.* number of dip-pad-dry steps.

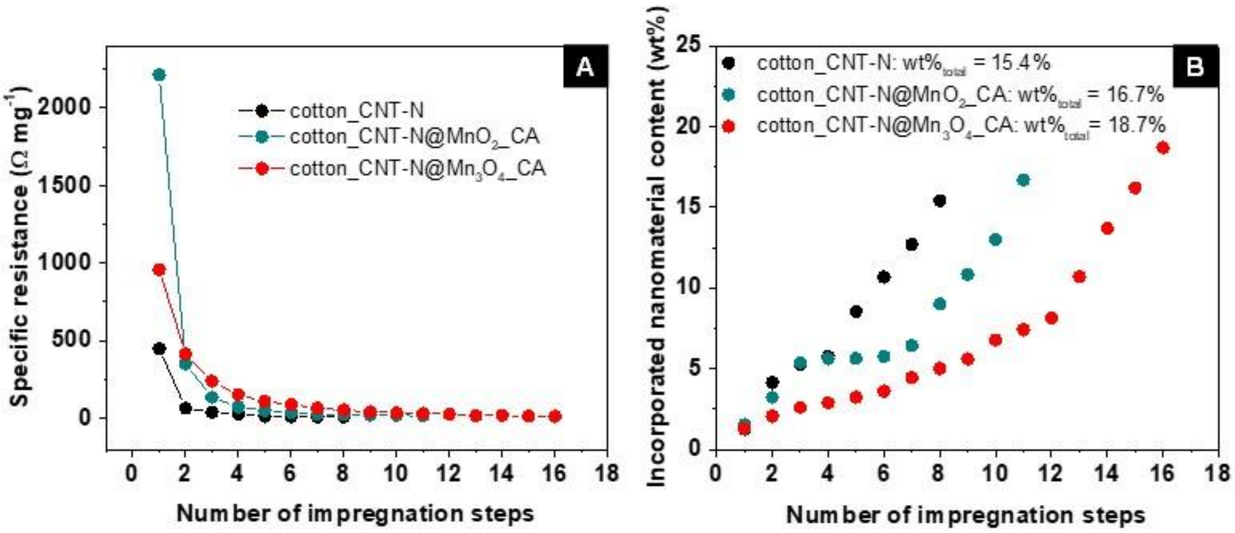

Figure S6. SEM micrograph (obtained in backscattered electrons mode) of (A) cotton_CNT-N@Mn_3O_4_CA, and the respective (B) carbon, (C) oxygen and (D) manganese elemental mapping analysis.

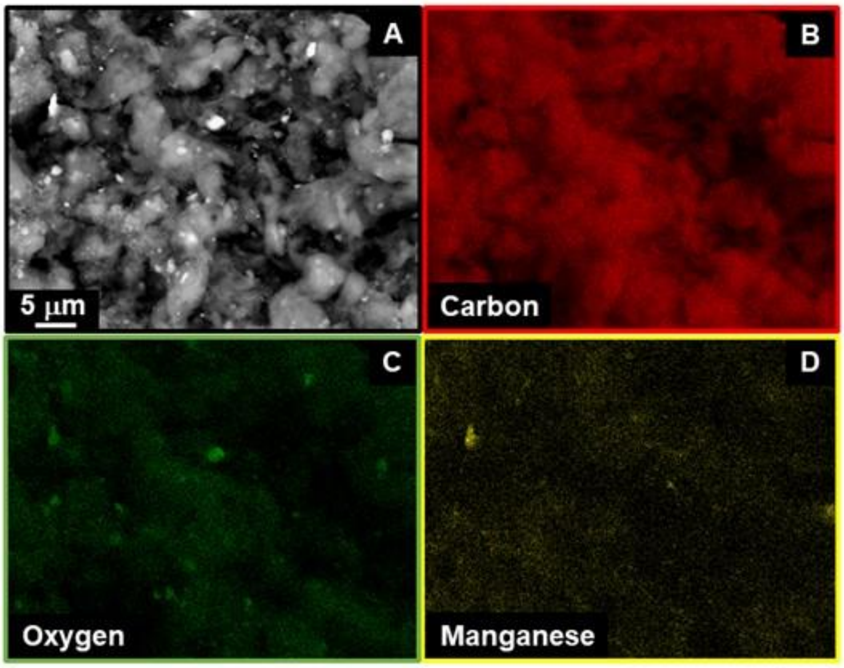

Figure S7. (A) Nyquist plots, (B) and (C) cyclic voltammograms at 1.0 mV s^{-1} in the potential window of -1.0 V to +1.0 V, (D) GCD curves and (E) charge/discharge curves at 0.1 mA cm^{-2} of the symmetric textile devices: CNT-N//CNT-N, CNT-N@MnO_2_CA//CNT-N@MnO_2_CA and CNT-N@Mn_3O_4//CNT-N@Mn_3O_4_CA.

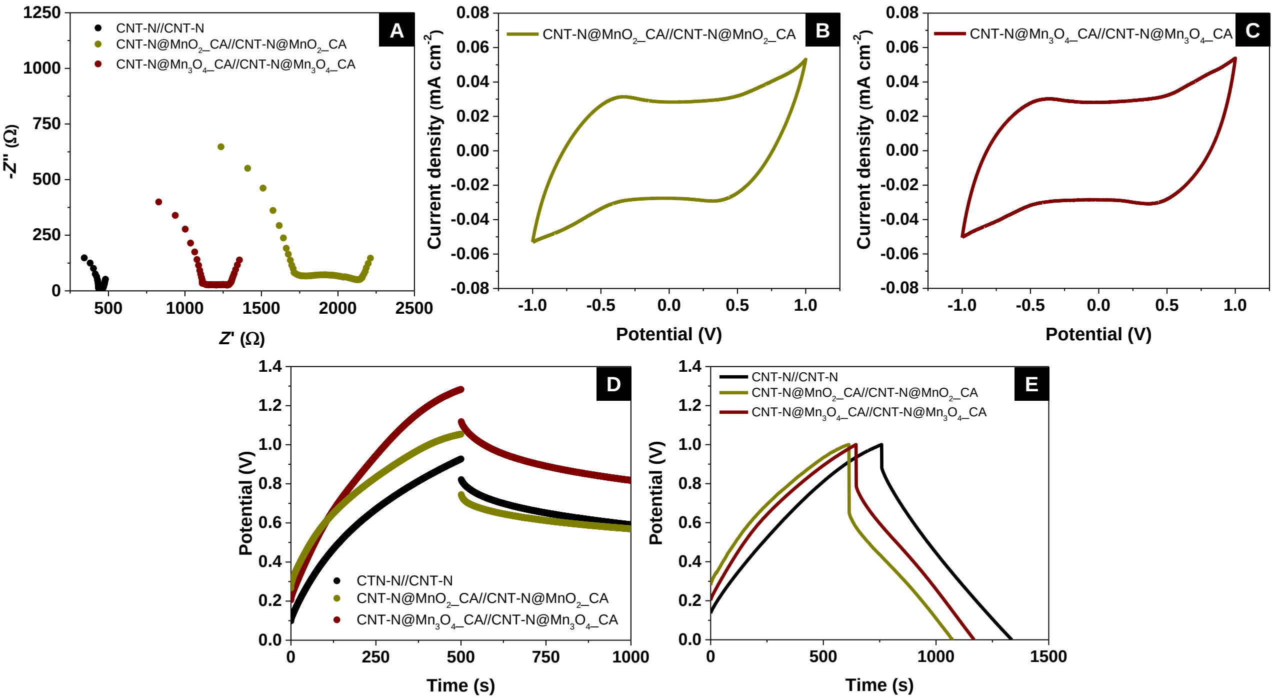

Figure S8. Cyclic voltammograms at 1 mV s^{-1} and different potential windows of the CNT-N-based textile SCs to determine the maximum potential window of each device: (A) CNT-N//CNT-N, (B) +CNT-N//CNT-N@MnO_2_CA− and (C) +CNT-N//CNT-N@Mn_3O_4_CA−.

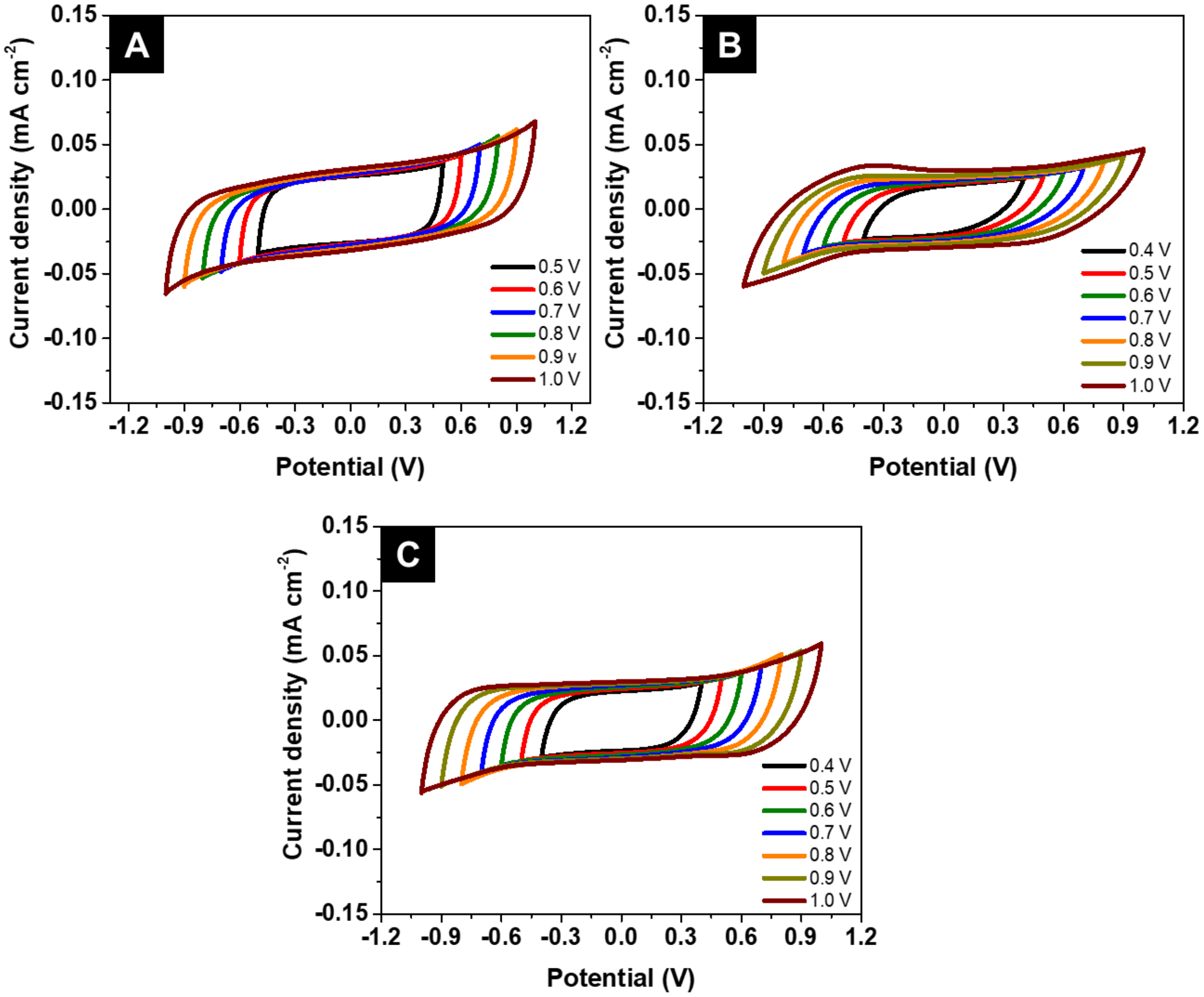

Figure S9. Cyclic voltammograms of the asymmetric textile SCs at 1.0 mV s^{-1} in the potential window of -1.0 V to +1.0 V: (A) +CNT-N//CNT-N@MnO_2_CA– and (B) +CNT-N//CNT-N@Mn_3O_4_CA–.

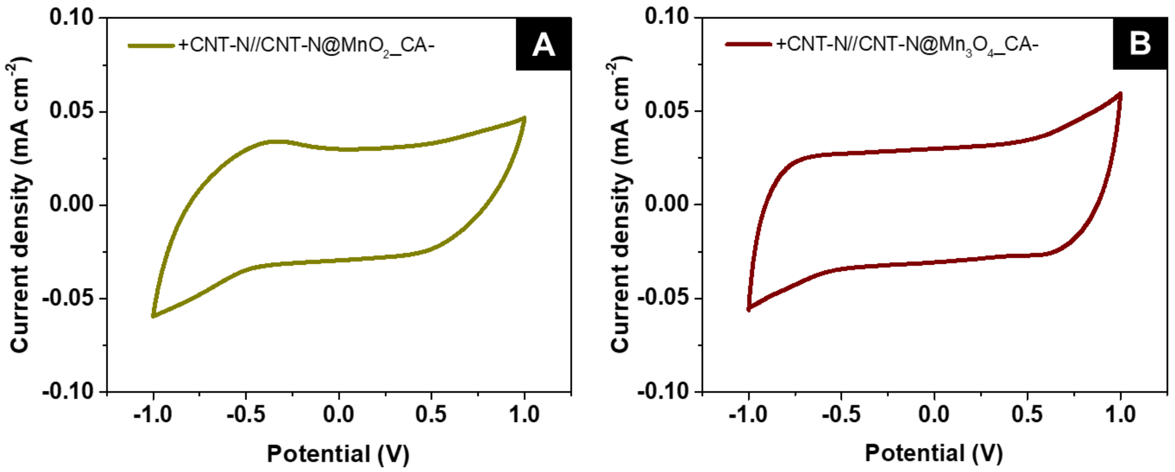

Figure S10. Charge and discharge curves at different current densities of 0.10, 0.15 and 0.20 mA cm^{-2} for (A) CNT-N//CNT-N, (B) +CNT-N//CNT-N@MnO_2_CA– and (C) −CNT-N//CNT-N@Mn_3O_4_CA+.

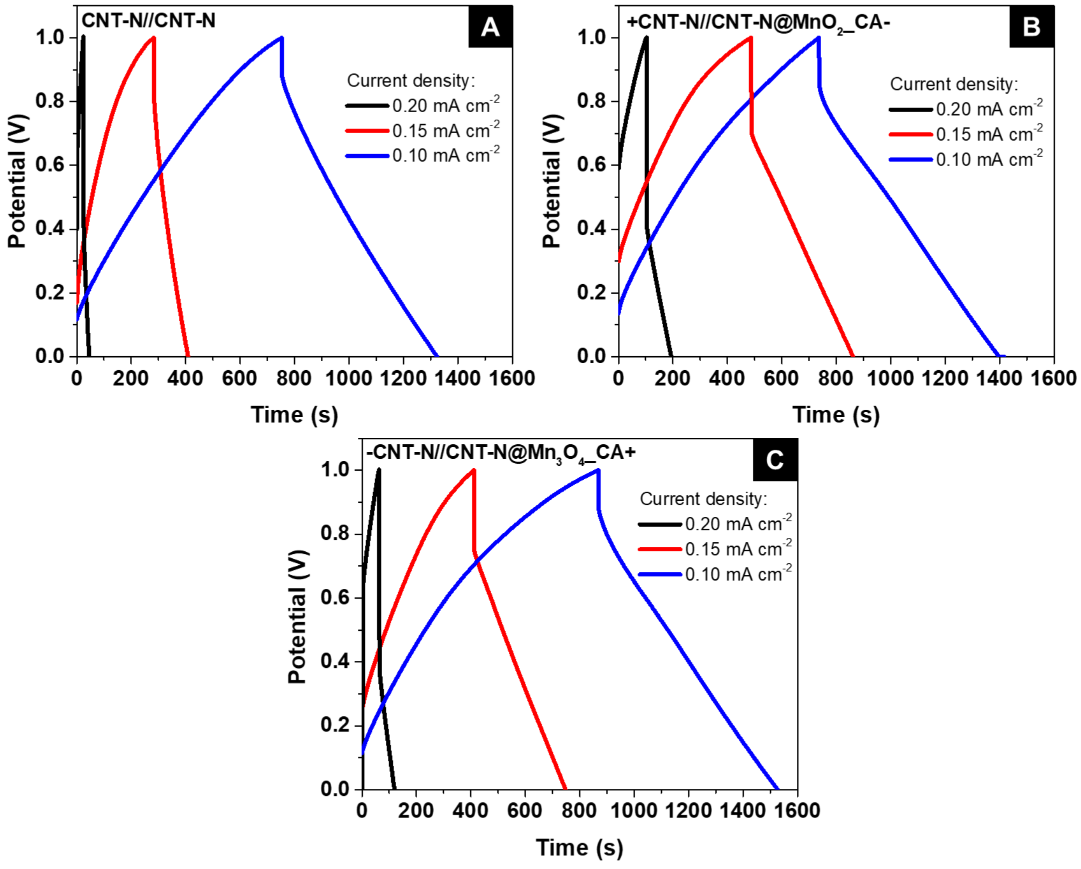

Figure S11. Cycling stability of (A) CNT-N//CNT-N, (B) –CNT-N//CNT-N@MnO_2_CA+ and (C) –CNT-N//CNT-N@Mn_3O_4_CA+, at 10 mV s^{-1}, for 8000 cycles.

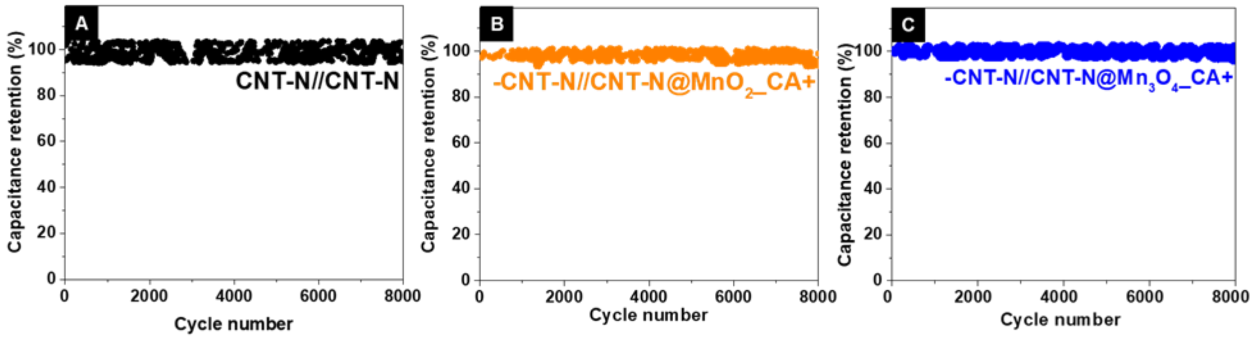

Figure S12. (A) X-ray diffractograms and (B) Raman spectra acquired with a $\lambda = 532$ nm laser of the pristine cotton fabric, CNT-N-based nanomaterials and textile electrodes before the assembly and in the assembled devices after the cycling tests.

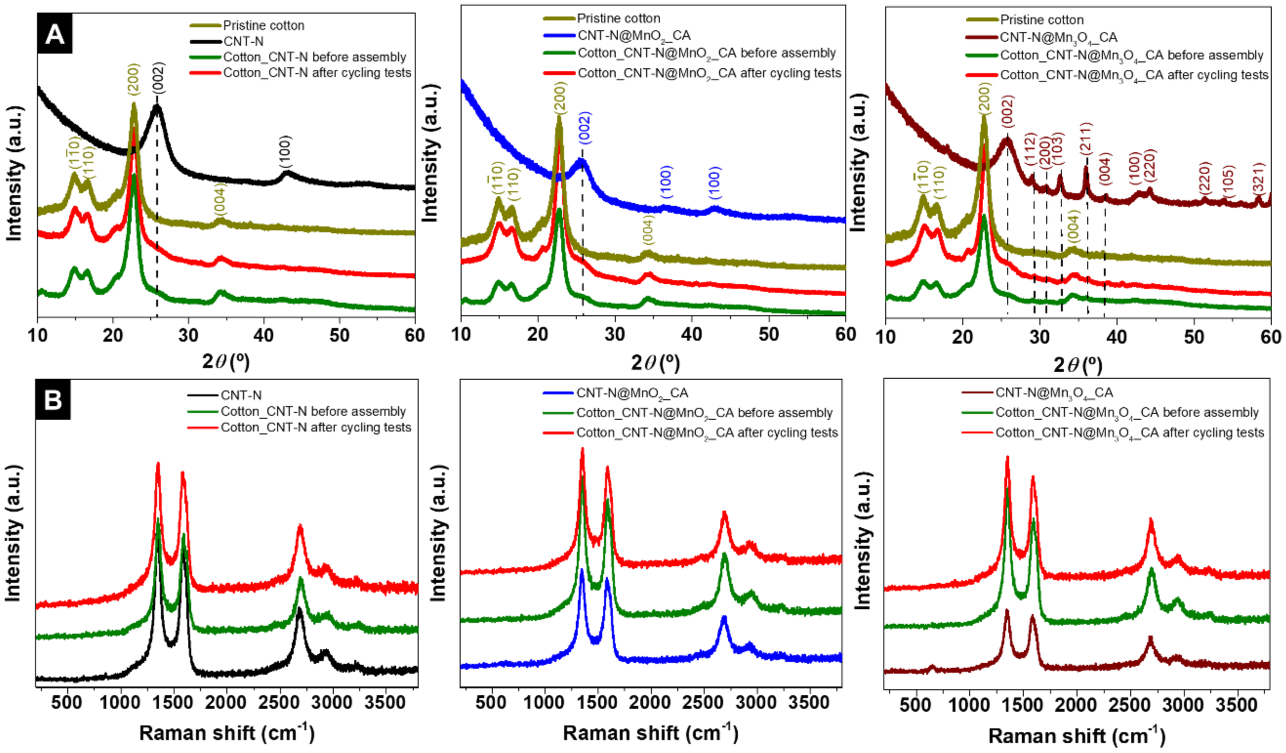

Figure S13. SEM images of the electrode and electrode/electrolyte interfacial sections of the symmetric CNT-N//CNT-N and asymmetric textile SCs after the cycling tests: (C) CNT-N textile electrode region, (D) CNT-N@MnO_2_CA textile electrode region and (E) CNT-N@Mn_3O_4_CA textile electrode region, with 150× (left), 5000× (middle) and 50000× (right) magnification.

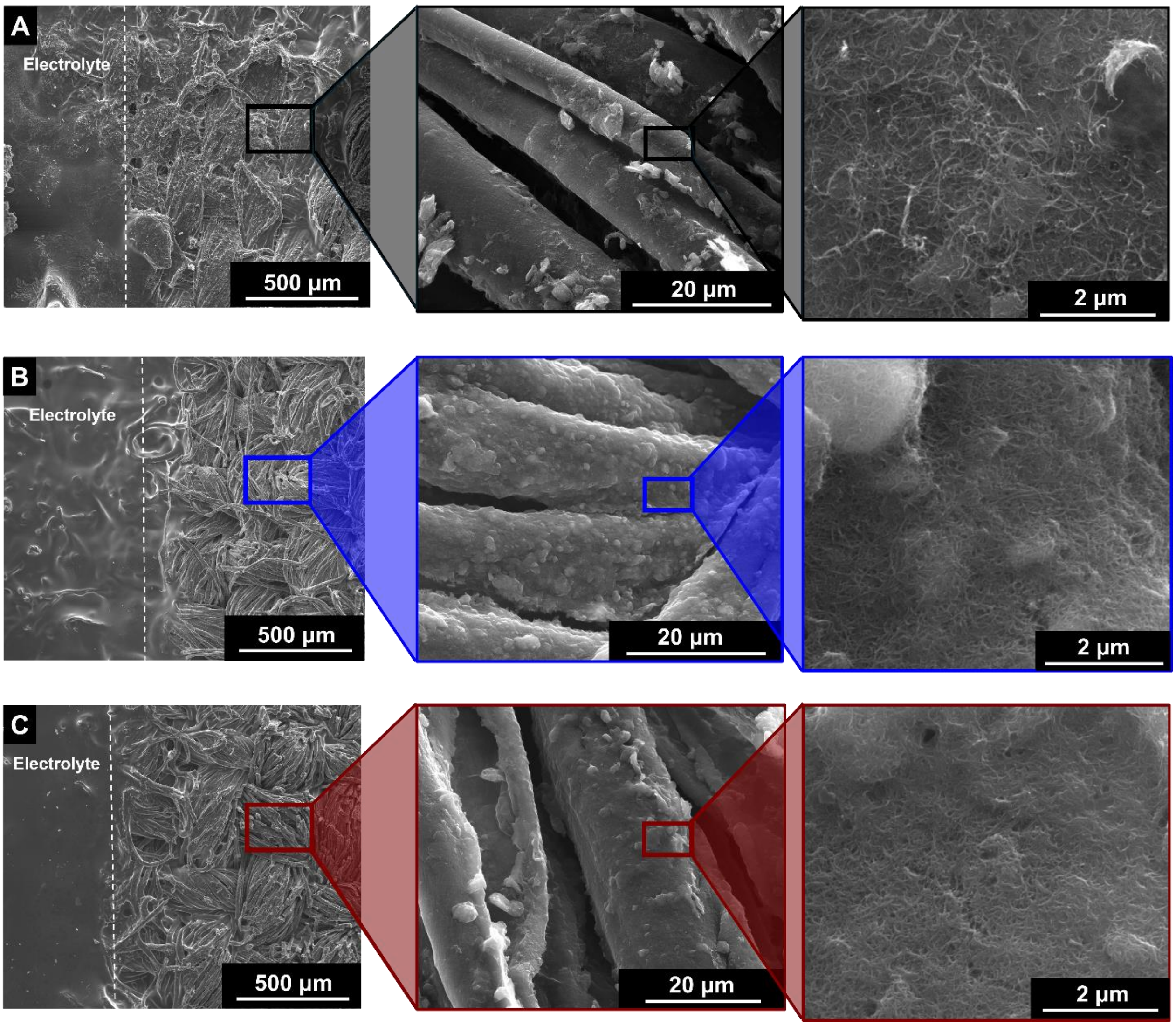

Figure S14. Electrochemical performance of two asymmetric devices of CNT-N//CNT-N@Mn_3O_4_CA connected in series or in parallel: (A) GCD curves at a current density of 0.10 mA cm^{-2} and (B) *i*-*V* cycles at 1 mV s^{-1}.

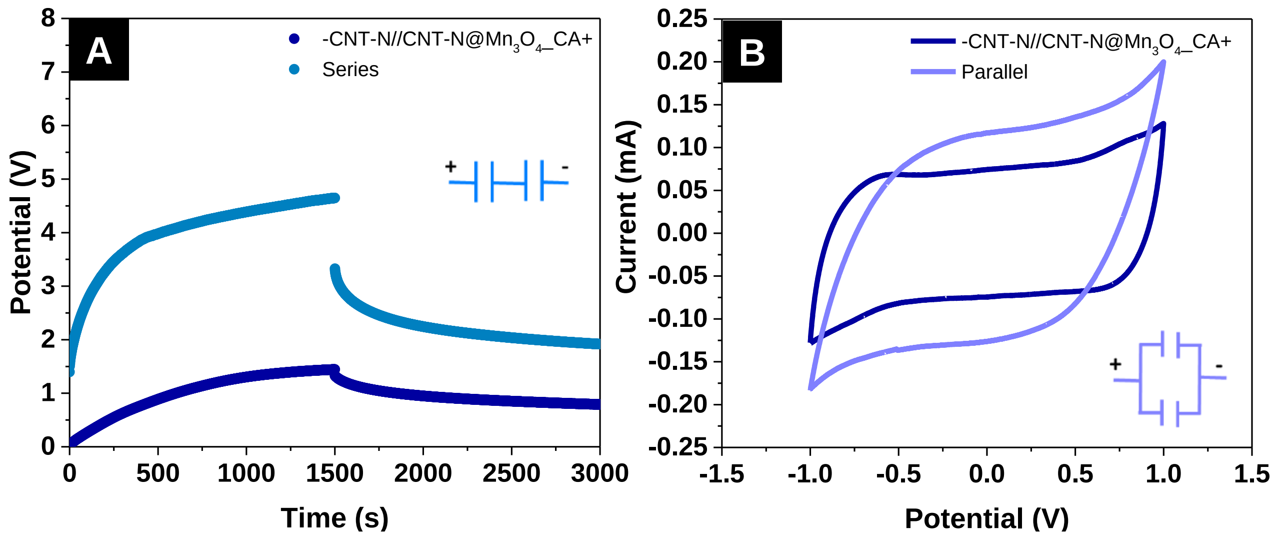

References

(S1) Li, Z.; Tian, M.; Sum, X.; Zhao. H.; Zhu, S.; Zhang, X. Flexible all-solid planar fibrous cellulose nonwoven fabric-based supercapacitor via capillarity-assisted graphene/MnO_2 assembly. *J. Alloys Compd.* **2019**, *782*, 986–994.

(S2) Wan, C.; Jiao, Y.; Liang, D.; Wu, Y.; Li J. A high-performance, all-textile and spirally wound asymmetric supercapacitors based on core–sheath structured MnO_2 nanoribbons and cotton-derived carbon cloth. *Electrochim. Acta* **2018**, *285*, 262–271.

(S3) Xang, P.; Xiao, X.; Li, Y.; Ding, Y.; Qiang, P.; Tan, X.; Mai, W.; Lin, Z.; Wu, W.; Li, T.; Jin, H.; Liu, P.; Zhou, J.; Wong, C. P.; Wang, Z. L. Hydrogenated ZnO Core–Shell Nanocables for Flexible Supercapacitors and Self-Powered Systems. *ACS Nano* **2013**, *7*, 2617–2626.

(S4) Jiang, H.; Zhou, C.; Yan, X.; Miao, J.; You, M.; Zhu, Y.; Li, Y.; Zhou, W.; Cheng, X. Effects of various electrolytes on the electrochemistry performance of Mn_3O_4/carbon cloth to ultra-flexible all-solid-state asymmetric supercapacitor. *J. Energy Storage* **2020**, *32*, 101898.

(S5) Fan, L.; Zhang, Yu.; Guo, Z.; Sun, B.; Tian, D.; Feng, Y.; Zhang, N.; Sun, K. Hierarchical Mn_3O_4 Anchored on 3D Graphene Aerogels via C-OMn Linkage with Superior Electrochemical Performance for Flexible Asymmetric Supercapacitor. *Chem. Eur. J.* **2019**, *26*, 9314–9318.

(S6) Dong, L.; Xu, C.; Li, Y.; Wu, C.; Jiang, B.; Yang, Q.; Zhou, E.; Kang, F.; Yang, Q.-H. Simultaneous Production of High-Performance Flexible Textile Electrodes and Fiber Electrodes for Wearable Energy Storage. *Adv. Mater.* **2016**, *28*, 1675–1681.

(S7) Kim, J. H.; Lee, J. M.; Park, J. W.; Kim, K. J.; Kim, S. J. MnO_2/PtNP Embedded Wet-Spun Fiber Supercapacitors. *Adv. Mater. Technol.* **2018**, *3*, 1800184.

(S8) Zhao, J.; Ma, Z.; Qiao, C.; Fan, Y.; Qin, X.; Shao, G. Spectroscopic Monitoring of the Electrode Process of MnO_2@rGO Nanospheres and Its Application in High-Performance Flexible Micro-Supercapacitors. *ACS Appl. Mater. Interfaces* **2022**, *14*, 34686–34696.

(S9) He, J.; Yang, D.; Li, H.; Cao, X.; Kang, L.; He, X.; Jiang, R.; Sun, J.; Lei, Z.; Liu, Z. Mn_3O_4/RGO/SWCNT hybrid film for all-solid-state flexible supercapacitor with high energy density. *Electrochim. Acta* **2018**, *283*, 174–182.
